# An advanced method for the release, enrichment and purification of high-quality *Arabidopsis thaliana* rosette leaf trichomes enables profound insights into the trichome proteome

**DOI:** 10.1186/s13007-021-00836-0

**Published:** 2022-01-28

**Authors:** Jan W. Huebbers, Kim Büttgen, Franz Leissing, Melissa Mantz, Markus Pauly, Pitter F. Huesgen, Ralph Panstruga

**Affiliations:** 1 Unit of Plant Molecular Cell Biology, Institute for Biology I, RWTH Aachen University, Worringerweg 1, 52056 Aachen, Germany; 2 Institute for Plant Cell Biology and Biotechnology, Heinrich-Heine-University Düsseldorf, Universitätsstr. 1, 40225 Düsseldorf, Germany; 3 Central Institute for Engineering, Electronics and Analytics, ZEA-3, Forschungszentrum Jülich, Jülich, Germany; 4 Cologne Excellence Cluster on Cellular Stress Responses in Aging-Associated Diseases (CECAD) Medical Faculty and University Hospital, University of Cologne, Cologne, Germany; 5 Institute of Biochemistry, Department for Chemistry, University of Cologne, Cologne, Germany

**Keywords:** Leaf trichome, *Arabidopsis thaliana*, Proteome, Cell wall analysis, Density gradient centrifugation, Histochemical staining

## Abstract

**Background:**

Rosette leaf trichomes of *Arabidopsis thaliana* have been broadly used to study cell development, cell differentiation and, more recently, cell wall biogenesis. However, trichome-specific biochemical or -omics analyses require a proper separation of trichomes from residual plant tissue. Thus, different strategies were proposed in the past for trichome isolation, which mostly rely on harsh conditions and suffer from low yield, thereby limiting the spectrum of downstream analyses.

**Results:**

To take trichome-leaf separation to the next level, we revised a previously proposed method for isolating *A. thaliana* trichomes by optimizing the mechanical and biochemical specifications for trichome release. We additionally introduced a density gradient centrifugation step to remove residual plant debris. We found that prolonged, yet mild seedling agitation increases the overall trichome yield by more than 60% compared to the original protocol. We noticed that subsequent density gradient centrifugation further visually enhances trichome purity, which may be advantageous for downstream analyses. Gene expression analysis by quantitative reverse transcriptase-polymerase chain reaction validated a substantial enrichment upon purification of trichomes by density gradient centrifugation. Histochemical and biochemical investigation of trichome cell wall composition indicated that unlike the original protocol gentle agitation during trichome release largely preserves trichome integrity. We used enriched and density gradient-purified trichomes for proteomic analysis in comparison to trichome-depleted leaf samples and present a comprehensive reference data set of trichome-resident and -enriched proteins. Collectively we identified 223 proteins that are highly enriched in trichomes as compared to trichome-depleted leaves. We further demonstrate that the procedure can be applied to retrieve diverse glandular and non-glandular trichome types from other plant species.

**Conclusions:**

We provide an advanced method for the isolation of *A. thaliana* leaf trichomes that outcompetes previous procedures regarding yield and purity. Due to the large amount of high-quality trichomes our method enabled profound insights into the so far largely unexplored *A. thaliana* trichome proteome. We anticipate that our protocol will be of use for a variety of downstream analyses, which are expected to shed further light on the biology of leaf trichomes in *A. thaliana* and possibly other plant species.

**Supplementary Information:**

The online version contains supplementary material available at 10.1186/s13007-021-00836-0.

## Background

Trichomes are fine outgrowths or appendages of plant epidermal cells. When fully differentiated, they can be uni- or multicellular, straight or branched, and either alive or dead. Non-glandular trichomes like those present on rosette leaves of the model plant *Arabidopsis thaliana* (thereafter for simplicity referred as “trichomes”) protect plants against water loss, ultraviolet (UV) radiation and herbivore attack [1]. *A. thaliana* trichomes are single-celled, up to 500 µm in height, subject to endoreduplication and typically have three or four branches [2, 3]. Due to their morphology, exposed position on the leaf surface and extraordinary size for a single cell, *A. thaliana* trichomes became a popular model in plant research [4, 5]. The role of trichomes as a paradigm for cell development and differentiation was spurred by the finding that the genetic, molecular and cell-biological processes involved in trichome development are frequently not trichome-specific but apply to the cellular activities of many tissues [4]. Furthermore, the dispensable nature of trichomes, at least under laboratory conditions, featured the study of mutants depicting deficiencies in trichome development. For example, mutant studies using *A. thaliana* leaf trichomes identified transcriptional regulators like GLABRA (GL1, GL2, GL3), TRANSPARENT TESTA GLABRA (TTG1, TTG2) and ENHANCER OF GLABRA3 (EGL3), which interplay in cell fate determination and trichome morphogenesis [6–11]. Furthermore, genes associated with gibberellic acid homeostasis such as *REPRESSOR OF GA* (*RGA*) and *GLABROUS INFLORESCENCE STEMS* (*GIS*) as well as proteins involved in epigenetic modifications like the histone chaperone CHROMATIN ASSEMBLY FACTOR-1(CAF-1) and the *N*^6^-methyladenosine reader protein EVOLUTIONARILY CONSERVED C-TERMINAL REGION2 (ECT2) were linked to trichome development [12–15]. The contribution of these components emphasizes the complexity of cell differentiation, involving phytohormones, transcription factors and epigenetic modifications. Especially the identification and functional analysis of transcription factors like GL and TTG in the late 1990s led to the proposal of a six-step model for trichome development in *A. thaliana* including (i) initiation and cell enlargement, (ii) polar growth, (iii) branching, (iv) branch growth, (v) diffuse growth and (vi) cell wall maturation [4, 5, 16]. In the last decade, the cell wall maturation step during trichome development was discovered as a model to study aspects of cell wall biogenesis in *A. thaliana* [2, 17, 18]. A unique callose deposition pattern in *A. thaliana* trichomes was described by Kulich et al. [18]. The authors of this study observed homogenous callose distribution in the apical trichome areas whereas no callose was deposited in the basal trichome regions. The boundary between apical and basal trichome zones was found to be formed by a callose-rich ring structure referred to, honoring its discoverer, as the Ortmannian ring [18].

Due to their eminent role as a model in various aspects of plant biology and their exposed presence on the surface of plant organs, trichomes have become a popular target for global profiling approaches via various -omics techniques. These procedures nevertheless rely on suitable techniques for the enrichment and purification of the biological sample material. In an initial approach, trichomes were manually clipped off by forceps and used for an image-based DNA quantification relying on staining with 4′,6-diamidino-2-phenylindole (DAPI) [19]. Later, this kind of mechanical trichome release was modified by adding a freezing step in liquid nitrogen prior to trichome sampling, which resulted in a first yet limited proteomic dataset [20]. As an alternative approach, microcapillaries were deployed to collect trichome cell content, which then was used for gene expression profiling [21, 22], chip-based protein analysis [23] or metabolomics [24]. The most recent method for trichome isolation relies on a biochemical approach that was published by Marks and co-workers [25] and represents an improved variant of a method originally proposed by Zhang and Oppenheimer in 2004 [26]. Without previously fixing the leaves, the authors used a generic tabletop vortex mixer for the high-speed mixing of seedlings in the presence of the cation chelator ethylene glycol-bis(β-aminoethyl ether)-*N*,*N*,*N*′,*N*′-tetraacetic acid (EGTA) and glass beads. While EGTA was considered to weaken trichome-leaf interactions as described before [26], the glass beads provided a mechanical stimulus for trichome release. After mixing, trichomes and seedlings were separated by two filtration steps, and residual crude plant debris was removed manually using forceps. Though the overall recovery was not quantified, this approach appeared to provide a much higher trichome yield compared to all previously described methods. The sampled trichomes were used for histochemical and biochemical evaluation of cell wall characteristics as well as microarray-based analysis of the trichome transcriptome [25, 27].

Here, we present a streamlined method for the release, enrichment and purification of *A. thaliana* trichomes. Resting upon the original idea of using EGTA to weaken trichome-leaf interactions and the modifications proposed by Marks et al. [25], we used an extended period for seedling incubation in EGTA-containing buffer coupled with mild steady mixing on a magnetic stirrer in the presence of glass beads. We show that the extended incubation and mixing time yields substantially more trichomes compared to speed mixing using a tabletop vortex mixer while reducing experimental effort as well as disposable waste and increasing scalability. Furthermore, we included a density gradient centrifugation (DGC) step that further enhanced trichome purity by reducing crude plant debris as well as fine impurities without significantly impairing trichome yield. We subjected trichomes enriched with this protocol to histochemical staining, biochemical assessment of cell wall components and proteomic analysis. The latter revealed 223 proteins that are highly enriched in trichomes compared to a leaf reference proteome.

## Results

### Density gradient centrifugation enhances trichome purity

**Fig. 1:**
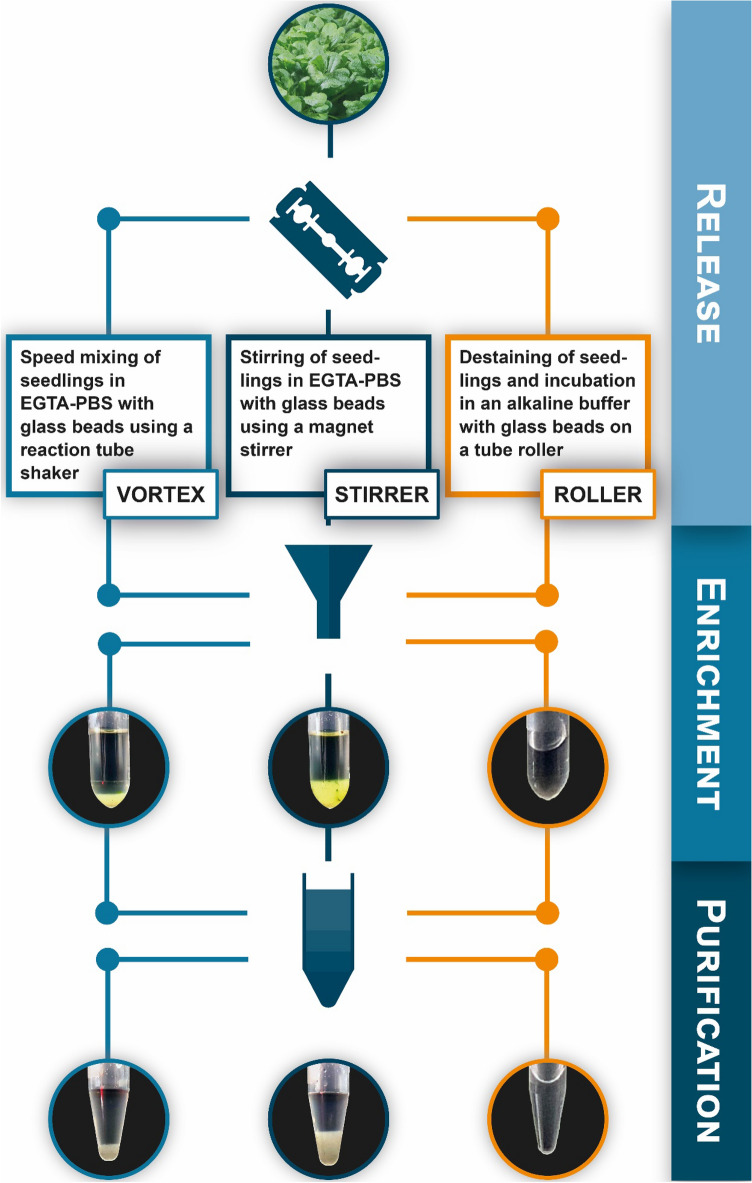
Workflow scheme for the isolation, enrichment and purification of *A. thaliana* trichomes by different methods. The diagram illustrates the main steps of the VORTEX, STIRRER and ROLLER procedures. For further details, see main text. The photographs shown at the intersection between the “Enrichment” and “Purification” steps exemplarily depict the outcome (trichomes as a pellet in a centrifugation tube) following filtration and centrifugation for each of the three release regimes. The photographs shown at the end of the “Purification” step exemplary illustrate the outcome (trichomes as a pellet in a centrifugation tube) following sucrose DGC for each of the three release regimes

**Fig. 2:**
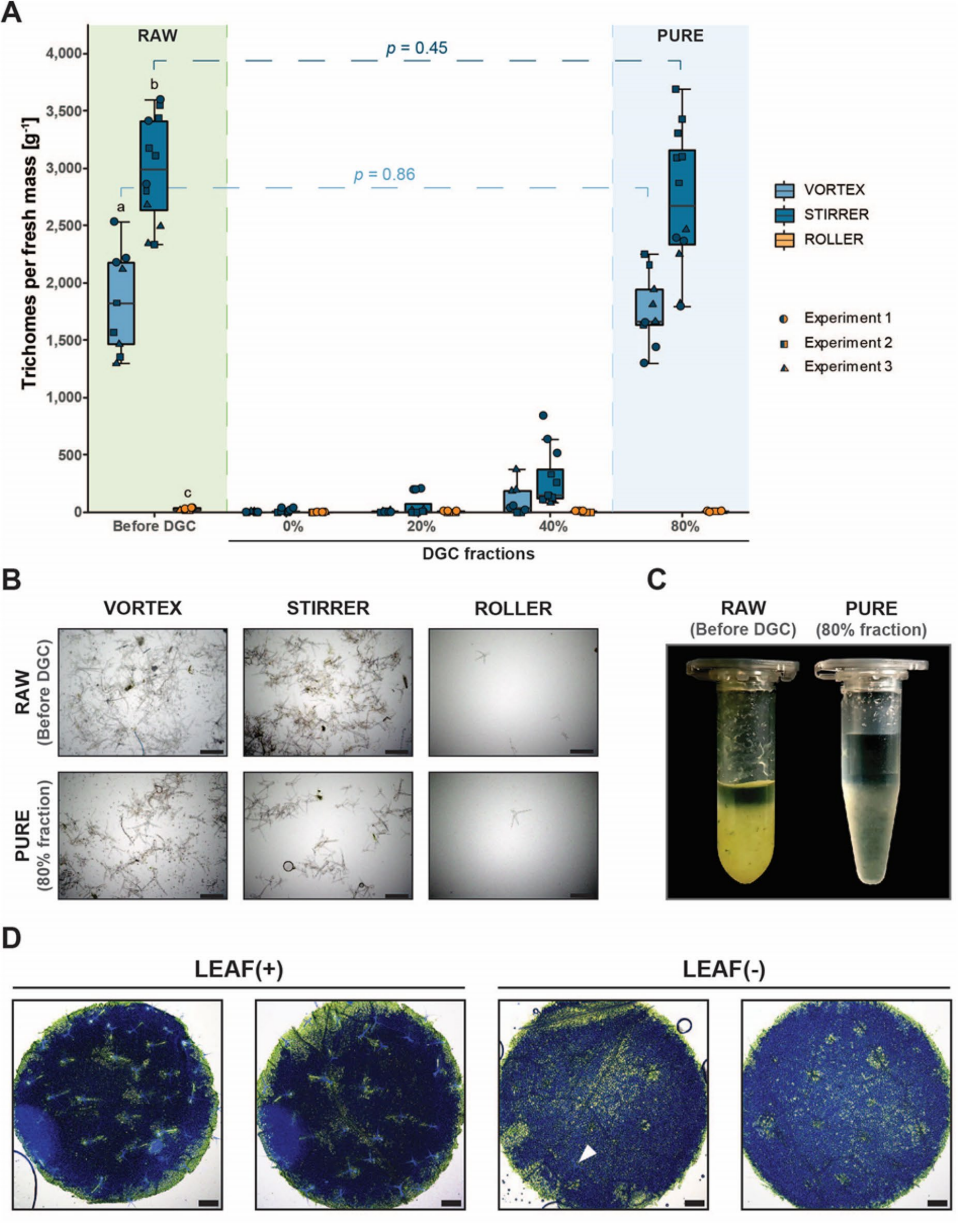
Evaluation of trichome quantity and quality after trichome release, enrichment and purification. **A** Quantification of trichomes recovered by the various purification regimes. The boxplot indicates the outcome of three experiments with three to six samples per experiment for the three methods. Letters indicate significance groups (Student’s *t*-test corrected with FDR, *α* = 0.01). Brackets and assigned *p*-values suggest differences in trichome number before DGC (RAW) and in the 80% fraction following DGC (PURE) for VORTEX and STIRRER samples (paired *t*-test). **B** Representative micrographs illustrating sample content and purity for the three procedures in RAW (before DGC) and PURE (after sucrose DGC) samples. Scale bars represent 500 µm. **C** Representative centrifugation tubes showing RAW (before DGC) and PURE (after sucrose DGC) trichome samples (STIRRER method). **D** Adaxial view of *A. thaliana* leaf discs before (LEAF(+)) and after (LEAF(−)) trichome release. Micrographs are merged pictures of brightfield and ultraviolet (UV) illumination with trichomes showing autofluorescence upon the latter. The arrowhead indicates a trichome that remained attached after the release procedure. Scale bars represent 500 µm

In order to improve trichome isolation, we modified the above described method developed by Marks et al. [25] both in terms of agitation (mechanical stimulus for trichome release) and buffer composition (chemical stimulus for trichome release). We compared the performance of those modifications to the original protocol concerning trichome yield, purity and integrity. Similar to the procedure reported by Marks and colleagues, *A. thaliana* (accession Col-0) wild-type seedlings were grown through the holes of a perforated metal plate and rapidly harvested (~ 200 g per batch) at 42 d after sowing by cutting them with a razor blade (Fig. 1, “Release”). After harvesting, the seedlings were rinsed thoroughly with tap water and trichome detachment was stimulated using one of the following procedures: (i) Transfer of seedlings to 50-mL reaction tubes filled with EGTA-PBS buffer as well as glass beads and mixing (3 × 30 s) on a reaction tube mixer (hereafter referred to as VORTEX method). (ii) Transfer of seedlings to a 500-mL beaker filled with EGTA-PBS buffer as well as glass beads and agitation (1.5 h) using a magnetic stirrer (hereafter referred to as STIRRER method). (iii) Transfer of seedlings to 50-mL reaction tubes filled with an alkaline buffer (pH 9.5) as well as glass beads and incubation (24 h) on a tube roller (hereafter referred to as ROLLER method) (Fig. 1, “Release”). After liberation, trichomes were enriched using two consecutive filtration steps as described before [25]. First, the liquid phase harboring potentially released trichomes was separated from the remaining *A. thaliana* tissue using four layers of screen door mesh. Afterwards, trichomes present in the excess liquid were captured using a cell strainer with a nominal pore size of 100 µm (Fig. 1, “Enrichment”). As in our hands a substantial amount of plant debris still co-purified with the detached trichomes in the liquid phase, we decided to include a subsequent density gradient centrifugation (DGC) step to increase sample purity further. For this purpose, we split the trichome samples equally and applied one-half of the enriched trichomes to discontinuous sucrose gradient centrifugation (Fig. 1, “Purification”). Whereas seedling-specific impurities gathered either at the interface of 20%/40% or 40%/80% (m/v) sucrose, trichomes passed the 80% sucrose layer and accumulated at the bottom of the 15-mL tube. After careful layer separation, by-sight evaluation of the purified trichomes revealed a considerable reduction of crude plant debris as well as greenish color (likely conditioned by pigment-containing tissue remnants) compared to trichome samples before DGC (Figs. 1, 2C).

### Magnetic stirring drastically increases trichome yield

For quantification of trichome yield by the three methods, we scored the number of intact trichomes in a volume of 20 µL using a brightfield microscope (Fig. 2A, B). Subsequently, we calculated the trichome count in a total sample volume of 1 mL by extrapolation and related it to the harvested seedling fresh mass. We found that the VORTEX method yielded an average trichome number of approx. 1800 g^−1^ before DGC (Fig. 2A). In comparison, the average amount of trichomes before DGC was about 99% lower (approx. 20 g^−1^) in case of the ROLLER method, suggesting a link between the presence of the EGTA-PBS buffer and trichome release efficiency. By contrast, trichome quantity before DGC was about 62% higher (approx. 3000 g^−1^) in samples processed by the STIRRER method compared to the VORTEX method, indicating a significant (Student’s *t*-test with false discovery rate (FDR) correction, *α* = 0.01) impact of the agitation setup on trichome release. We assessed the reproducibility of VORTEX- and STIRRER-based trichome release by calculating the coefficients of variation (CoVs) among the three independent experiments we carried out to score trichome yield prior to DGC (RAW; Fig. 2A). We found a similar high reproducibility for the VORTEX and STRIRRER method with comparably low CoVs of 0.22 and 0.14, respectively. Furthermore, we quantified the number of intact trichomes after DGC in each fraction of the discontinuous sucrose gradient. As suggested by visual inspection, the majority of trichomes (VORTEX: ~ 95%, STIRRER: ~ 89%, ROLLER: ~ 52%) gathered at the bottom of the reaction tube and were found in the 80% fraction after gradient separation (Fig. 2A). Though trichomes were also recovered from the 0%, 20% and 40% fractions of the sucrose gradient, paired *t*-test indicates no statistically significant difference in trichome counts before DGC and in the 80% fraction of VORTEX and STIRRER samples, suggesting no significant loss of trichomes during DGC. By contrast, a considerably higher trichome count in the 40% fraction of the STIRRER samples (approx. 300 g^−1^) compared to the same fraction of the VORTEX samples (approx. 100 g^−1^) may signify that the STIRRER method might affect the density of at least some of the purified trichomes. Whereas both trichomes and plant-specific impurities were rare in samples processed by the ROLLER method, evaluation by microscopy of VORTEX and STIRRER samples before DGC revealed a high degree of crude debris and fine particles (Fig. 2B). Especially the number of fine particles was visibly reduced after DGC in the 80% fractions of VORTEX and STIRRER samples. Furthermore, a comparison of reaction tubes harboring either trichome samples before DGC or trichomes recovered from the 80% fraction showed a visible reduction in greenish appearance and crude, foliaceous plant debris (Fig. 2C). Inspection of leaf discs prepared from *A. thaliana* seedlings before (LEAF(+)) and after (LEAF(−)) trichome detachment by brightfield microscopy suggests an almost complete release of trichomes by the STIRRER method (Fig. 2D). As a potentially cost-saving adaptation of the STIRRER protocol, we further explored whether EGTA could be replaced by the considerably cheaper chelating agent EDTA (ethylene diamine *N*,*N*,*N*′,*N*′-tetraacetic acid). We found no difference regarding trichome yield prior to DGC when carrying out the STIRRER method with either EGTA or EDTA. There was likewise no difference regarding trichome yield in the 0% and 40% fractions and even a somewhat higher yield with EDTA in the 80% fraction following DGC (Additional file 1: Fig. S1).

### Trichome release by magnetic stirring is applicable to other plant species

**Fig. 3:**
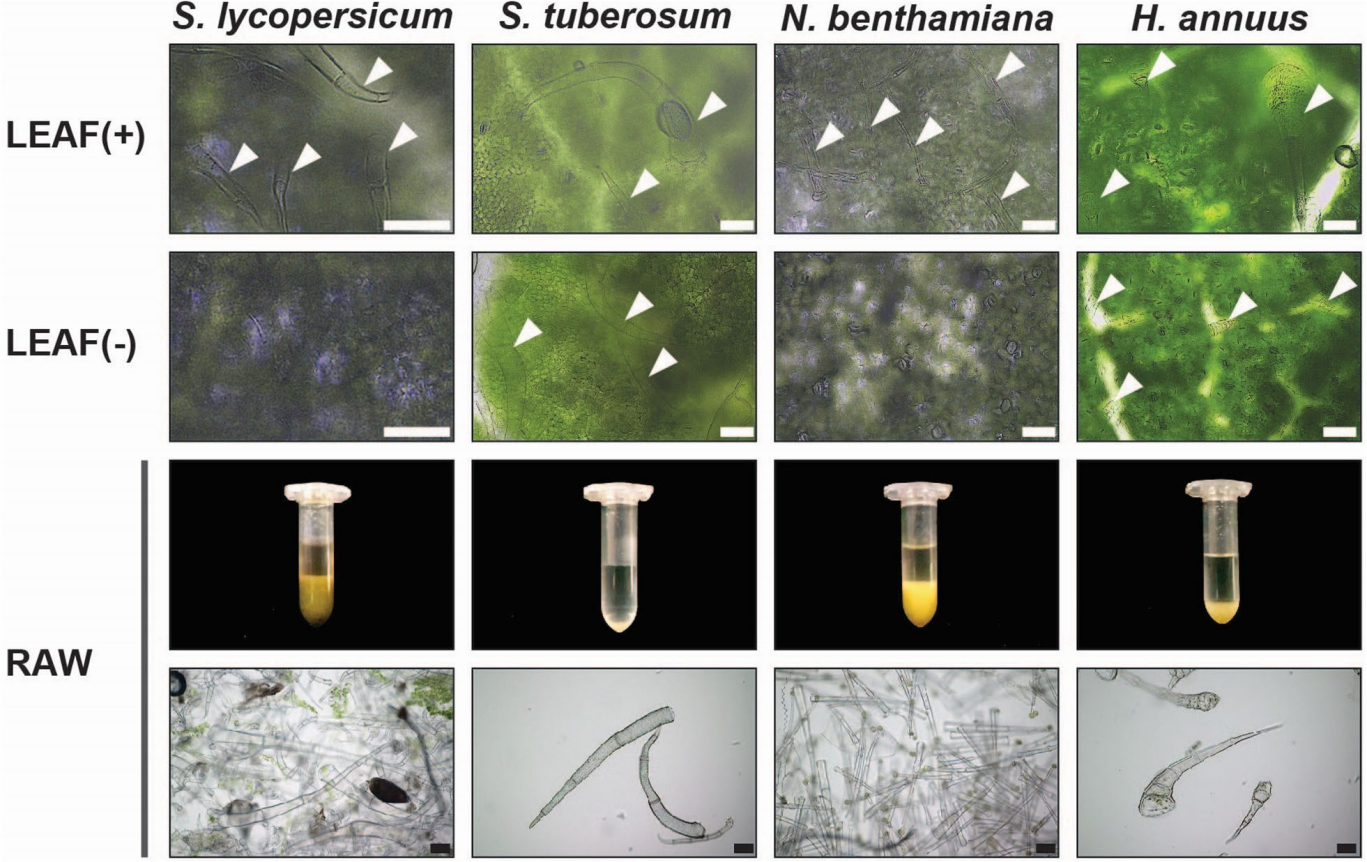
Isolation of trichomes from other plant species. Trichomes were released from *S. lycopersicum*, *S. tuberosum*, *N. benthamiana* and *H. annuus* by the STIRRER method and enriched as described for *A. thaliana*. Please note that due to the leaf size of these plants we reduced the amount of processed plant material to about 15 g per sample to ensure free floating of the leaves in the EDTA-PBS buffer. Micrographs show the adaxial side of leaves before (LEAF(+)) and after (LEAF(−)) trichome release as well as detached trichomes after enrichment (RAW). Arrowheads in the LEAF(−) micrographs mark trichomes that remained attached to the leaf surface. Reaction tubes show total trichome yield per plant species. Brightfield microscopy of enriched trichomes was performed with a 50 µL sample of the 1 mL trichome suspension. Scale bars represent 100 µm

Apart from *A. thaliana,* we tested if the STIRRER method is suitable for the isolation of leaf trichomes from *Solanum lycopersicum* (tomato), *S. tuberosum* (potato), *Nicotiana benthamiana* and *Helianthus annuus* (sunflower). For this purpose, we harvested leaf material from these plant species and carried out trichome release and enrichment as described before using EDTA-PBS buffer (Fig. 1). Comparison of LEAF(+) and LEAF(−) samples by brightfield microscopy revealed similar to *A. thaliana* (Fig. 2D) a near complete loss of trichomes in LEAF(−) samples in the case of *S. lycopersicum* and *N. benthamiana* whereas in the event of *H. annuus* residual trichomes of the linear glandular type were retained (Fig. 3). Concerning *S. tuberosum*, inspection by microscopy revealed equal amounts of trichomes on the surface of LEAF(+) and LEAF(−) samples, indicative of incomplete trichome release. Accordingly, we observed the lowest amount of trichomes in RAW samples in the case of *S. lycopersicum* (Fig. 3). Likewise, the fresh mass (1.00 mg g^−1^) and dry mass (0.07 mg g^−1^) of trichomes released from *S. tuberosum* per harvested leaf mass was considerably lower than the respective values for the other plant species tested (Additional file 1: Table S1). Regarding *H. annuus*, we measured relative trichome yields of 3.89 mg g^−1^ for fresh trichomes and 0.28 mg g^−1^ for lyophilized trichomes, which was about half of the trichome yield gathered from *A. thaliana* rosette leaves under the same conditions (Additional file 1: Table S1). By contrast, the trichome yield from *S. lycopersicum* (fresh mass 18.33 mg g^−1^, dry mass 1.07 mg g^−1^) and *N. benthamiana* (fresh mass: 19.00 mg g^−1^, dry mass: 1.07 mg g^−1^) was approx. twice as high as for *A. thaliana* (Additional file 1: Table S1). Brightfield microscopy of trichome samples reflected the amounts indicated by visual inspection of the pellet in the reaction tubes (Fig. 3) and the measured trichome yields (Additional file 1: Table S1). Furthermore, the morphology of the isolated trichomes was in accordance with trichomes observed on the surface of LEAF(+) samples (Additional file 1: Fig. S2). For *S. lycopersicum*, we could identify four different types of trichomes (Additional file 1: Fig. S2A). Similarly, we identified two different trichome types for *N. benthamiana* (Additional file 1: Fig. S2B).

### Trichome release by magnetic stirring reduces Toluidine blue O staining efficiency

**Fig. 4:**
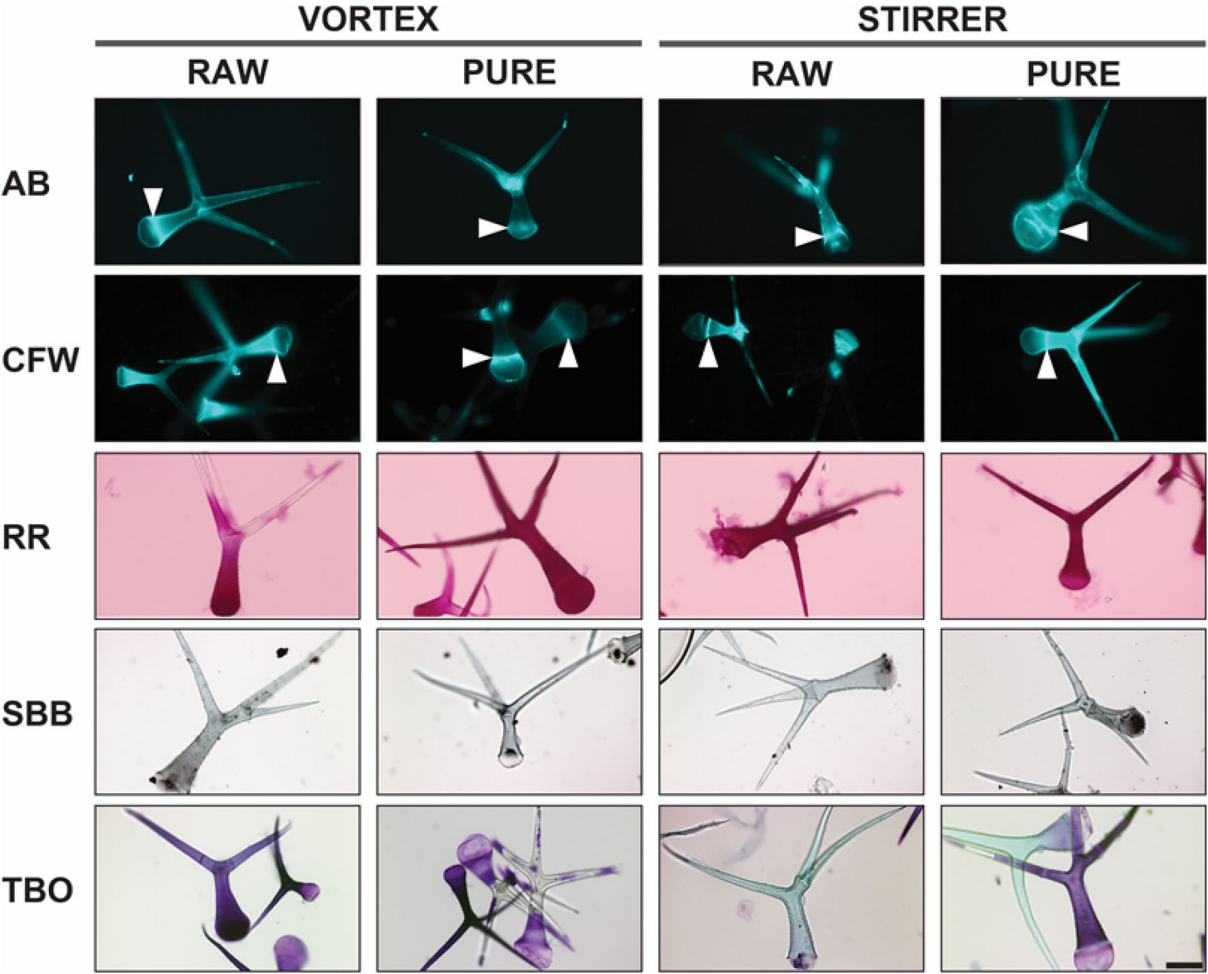
Histochemical staining of *A. thaliana* trichomes. Trichomes released and enriched by the various regimes (VORTEX or STIRRER) were subjected to histochemical staining and observation by epifluorescence (Aniline Blue (AB) and Calcofluor White (CFW)) or brightfield microscopy (Ruthenium Red (RR), Suden Black B (SBB) and Toluidine Blue O (TBO)). Arrowheads mark the Ortmannian ring in AB- and CFW-stained specimens. The size bar (STIRRER, PURE, TBO) equals 100 µm

We applied different histochemical stains to our isolated trichomes to examine how the release strategy (VORTEX or STIRRER) and/or DGC affect(s) the composition of the trichome cell walls (Table 1). We used Aniline Blue and Calcofluor White to visualize β-linked polysaccharides under ultraviolet (UV) illumination (epifluorescence microscopy). Whereas Calcofluor White visualizes both 1,3-β- and 1,4-β-glucans, Aniline Blue specifically probes the 1,3-β-linked cell wall polymer callose [28–30]. For both stains, we did not observe distinct differences that can be traced back to either the release method or DGC purification among our trichome samples (Fig. 4). Regarding Aniline Blue staining, we observed a strong fluorescence signal at the interface between the basal and apical trichome segments as well as in the branching area of the trichomes. Compared to Aniline Blue, the Calcofluor White signal was more homogenously distributed across the whole trichome area in most samples. Furthermore, Calcofluor White staining led to a better visualization of the Ortmannian ring than Aniline Blue staining (Fig. 4). Besides Aniline Blue and Calcofluor White, we also used three colorimetric stains including (i) Ruthenium Red for pectin visualization, (ii) Sudan Black B for lipid detection and (iii) Toluidine Blue O, which is described to stain pectic/polygalacturonic acid pinkish purple and poly-aromatic substances such as lignin blue or greenish-blue [31]. After Ruthenium Red staining, all trichomes showed a homogenously distributed red color under brightfield illumination, pointing to a generally even pectin distribution. Furthermore, we observed reddish congregations at the trichome surface of unpurified trichomes, which were considerably reduced in the purified trichome samples, suggesting these corresponded to pectin-containing debris sticking to the unpurified trichomes. Concerning Sudan Black B, all trichomes showed a black staining at the trichome surface and black congregations inside the trichomes. Upon incubation in Toluidine Blue O solution for 5 min, trichomes released by the VORTEX method were predominantly stained pinkish purple, indicative of polygalacturonic acid, whereas most trichomes released by the STIRRER method remained either transparent or were weakly stained (Fig. 4; Additional file 1: Fig. S3A). After an incubation of 1 h in Toluidine Blue O, instead, trichomes released by both the VORTEX and the STIRRER method appeared pinkish purple (Additional file 1: Fig. S3A). Since no evidence for lignification was found upon Toluidine Blue O staining of our isolated trichomes, we also stained trichomes released by the STIRRER method with potassium permanganate under acidic conditions (Mäule reaction). Following Mäule reaction, purified and non-purified trichomes showed a homogenous brown coloration (Additional file 1: Fig. S3B), suggesting an overall low level of lignification. However, the Ortmannian ring was stained in a more intense brown color compared to the rest of the cell in some trichomes, suggesting it occasionally exhibits a larger degree of lignification than the other trichome regions.

**Table 1:** Histochemical staining patterns of trichomes isolated by various methods

Stain	VORTEX		STIRRER	
	RAW	PURE	RAW	PURE
Aniline Blue	Distinct signals at the interface between the basal and apical trichome regions (OR) as well as in the branching area			
Calcofluor White	Homogeneous staining of the whole trichome and occasionally distinct signals at the interface between the basal and apical trichome regions (OR) as well as at the borderlines between stalk and branches			
Ruthenium Red	Homogeneous red coloration			
	Reddish congregations at the trichome surface		Reddish congregations at the trichome surface	
Sudan Black B	Black staining of trichome surface and dark congregations inside trichomes			
Toluidine Blue O	Pinkish-purple staining, either homogeneous or in a speckled pattern		Largely transparent or weakly stained after 5 min; pinkish-purple staining after 1 h	
Mäule reaction	-		Homogenous brown staining and occasionally visualization of the OR	

OR: Ortmannian Ring

### Trichome release by magnetic stirring reduces variation in the quantification of wall monosaccharides

**Fig. 5:**
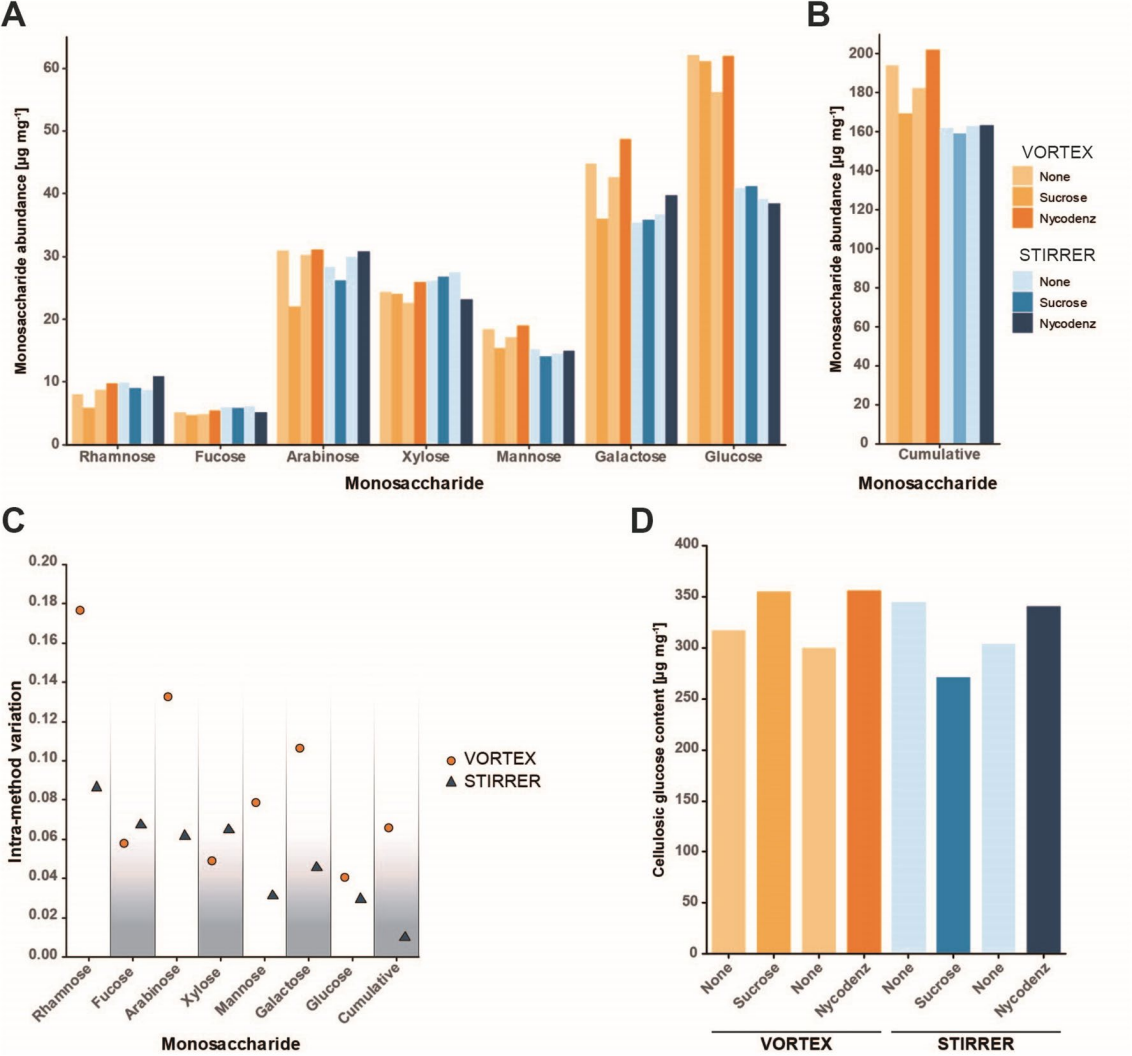
Analysis of the cell wall composition of *A. thaliana* trichomes. The amount of cell wall matrix monosaccharides and crystalline cellulose in trichomes was determined. RAW samples and trichomes purified by a Nycodenz DGC were included, to assess the impact of residual glucose in trichome samples after sucrose DCG. Columns indicate the result of a single experiment. **A** Amount of cell wall monosaccharides in the alcohol insoluble residue (in mg) recovered by the indicated release and purification strategies. **B** Total amount of all matrix wall monosaccharides in the alcohol insoluble residue (in mg) of the various samples. **C** Intra-method variation of monosaccharide amounts among trichomes processed by the indicated methods. Dots represent the CoV of the four values presented for each method in panel A. **D** Amount of cellulosic glucose per trichome in the alcohol insoluble residue (in mg) recovered by the indicated release and purification strategies. Values in panels A, B and D are given per mg alcohol insoluble residue

Besides histochemical analyses, we aimed at quantifying the matrix monosaccharide composition and crystalline cellulose content of the purified trichomes. Since the sucrose used during DGC might distort glucose and hence also cellulose values in such an analysis, we tested the non-ionic iodinated benzoic acid derivative Nycodenz as an alternative gradient medium. Our comparison between sucrose and Nycodenz densities revealed that 60% (m/v) Nycodenz resembles the density of 80% (m/v) sucrose (Additional file 1: Fig. S4A). The densities of the layers of the Nycodenz gradient were adapted accordingly (Additional file 1: Fig. S4B). Subsequently, we compared the measured cellulose and matrix monosaccharide levels of samples enriched in a Nycodenz gradient to values measured in trichome cell walls purified with sucrose gradients and samples that were not purified by DGC. We did not observe any impact of the purification strategy (none, sucrose or Nycodenz gradient) on the individual or total monosaccharide composition (Fig. 5A, B). Regarding the release strategy (VORTEX or STIRRER), however, we found that the galactose and glucose levels were increased in trichomes processed by the VORTEX method compared to the STIRRER method, which was mirrored by an elevated total monosaccharide amount in the VORTEX samples (Fig. 5A, B). We also observed that for certain monosaccharides (rhamnose, arabinose, mannose, galactose) the CoV among trichomes released by the VORTEX method was about 50% higher compared to trichomes released by the STIRRER method (Fig. 5C). Furthermore, we assessed the cellulose content in our purified and non-purified trichomes but found no considerable impact of either the release method (VORTEX or STIRRER) or the purification strategy (none, sucrose or Nycodenz gradient) (Fig. 5D).

### Gene expression analysis and protein profiling of isolated trichomes

**Fig. 6:**
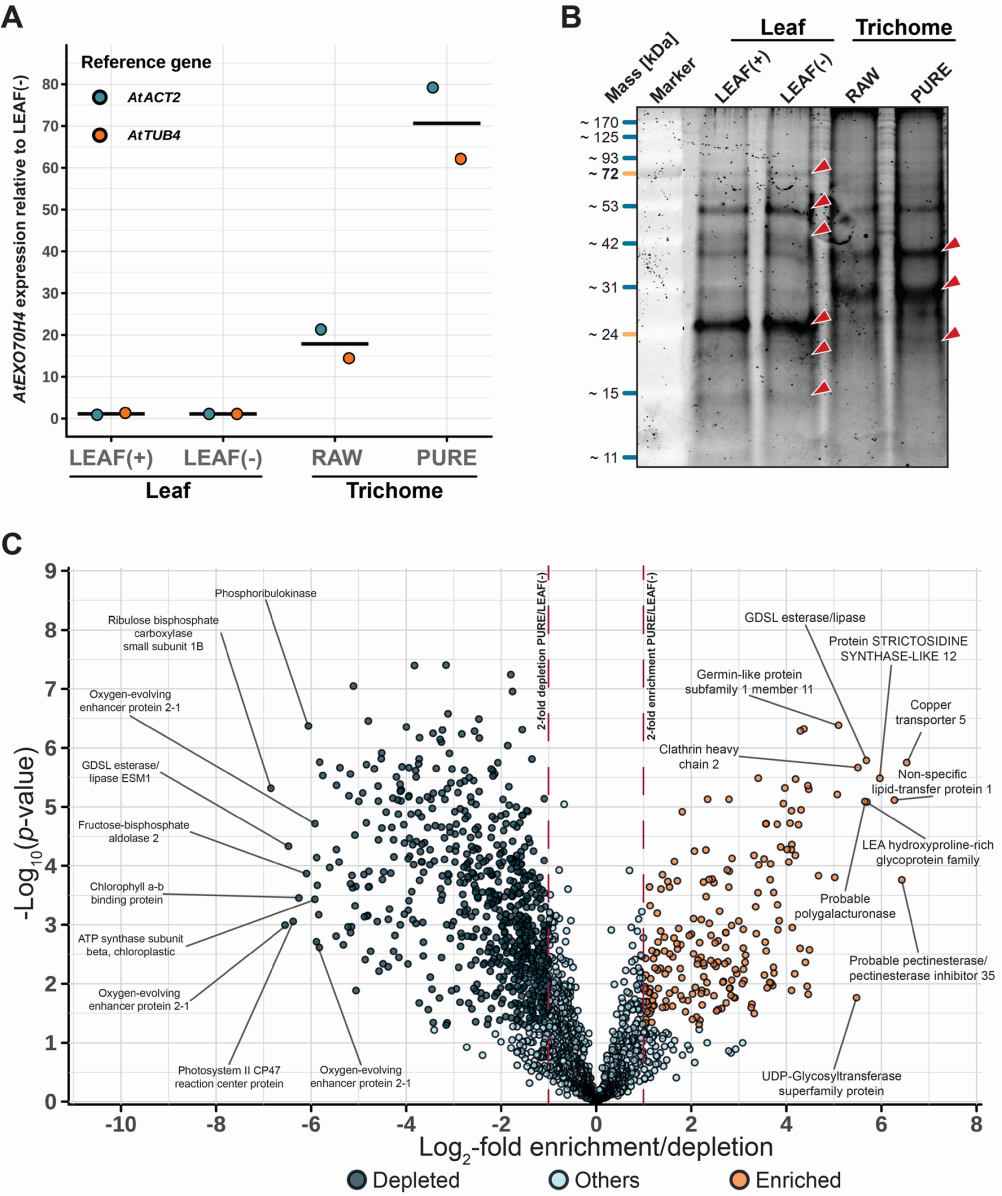
Comparison of *A. thaliana* leaf tissue and trichomes regarding *AtEXO70H4* expression and proteome composition. **A** Transcript abundance of *AtEXO70H4* in leaf and trichome samples. *AtEXO70H4* transcript levels were determined by qRT-PCR using the 2^−∆∆CT^ method. Dots represent the mean of three replicates in a single experiment relative to *AtACT2* or *AtTUB4* expression. Lines indicate the mean of *AtEXO70H4* expression regarding the two reference genes. **B** Total protein extracts of leaf and trichome samples after SDS-PAGE and SYPRO Ruby gel staining. Red arrowheads mark more intense signals upon comparison of LEAF(−) and PURE samples. **C** Proteins identified in trichome PURE samples referred to LEAF(−) samples depending on the respective *p*-values (Student’s *t*-test). Top ten enriched/depleted proteins were tagged by name. Dots represent proteins identified in at least three of four measured samples. Vertical dashed lines indicate the borders for two-fold enrichment or depletion. Coloration of the dots highlights proteins significantly and at least two-fold enriched or depleted in PURE compared to LEAF(−) samples. "Others" refers to proteins that were not assigned to one of the two categories. LEAF(+): *A. thaliana* leaves harboring trichomes, LEAF(−): Leaves after trichome release, RAW: Trichomes before DGC, PURE: Trichomes after sucrose DGC

Extending the above-described analysis of carbohydrate cell wall metabolites, we wondered whether the STIRRER method is also compatible with transcriptomic and proteomic downstream assays. For this purpose, we gathered four different sample types during the isolation process including (i) *A. thaliana* fresh leaves harboring trichomes (LEAF( +)), (ii) leaves disposed after STIRRER-mediated trichome release (LEAF(−)), (iii) STIRRER-isolated trichomes before sucrose DGC (RAW) and (iv) STIRRER-isolated trichomes recovered from the 80% fraction after sucrose DGC (PURE). Using these four sample types, we initially evaluated if the trichome quality is sufficient for RNA isolation and gene expression analysis. To this end, we extracted total RNA and carried out quantitative reverse transcriptase-polymerase chain reaction (qRT-PCR) analysis to exemplarily determine *AtEXO70H4* (*At3g09520*) transcript levels. *AtEXO70H4* encodes an EXO70 exocyst subunit that is required for proper callose deposition in trichomes [18]. It has been described as one of the 5% most highly expressed genes in trichomes while its transcript is essentially absent in most other cell types of rosette leaves under standard conditions [32] (http://bar.utoronto.ca/efp/cgi-bin/efpWeb.cgi). While we detected similarly low *AtEXO70H4* transcript levels in our leaf samples (LEAF( +), LEAF(-)), we found that the gene shows considerably higher transcript accumulation in RAW (mean(2^−∆∆CT^) = 21.3) and PURE (mean(2^−∆∆CT^) = 79.2) trichomes compared to LEAF(-) samples using *AtACT2* (*At3g18780*) and *AtTUB4* (*At5g44340*) as reference genes for normalization (Fig. 6A). Agarose gel electrophoresis and Sanger sequencing validated the purity and identity of all qRT-PCR products of this experiment (Figure S5).

As described above for RNA, we next extracted total proteins from two leaf (LEAF(+) and LEAF(−)) and two STIRRER-isolated trichome samples (RAW and PURE). After their separation by sodium dodecyl sulfate–polyacrylamide gel electrophoresis (SDS-PAGE) and SYPRO Ruby gel staining, we observed a distinctive band pattern in the case of RAW and PURE trichomes, which was characterized by two prominent signals linked to molecular masses of ~ 30 kDa and ~ 40 kDa (Fig. 6B). The intensity of both of these signals was considerably increased compared to the leaf samples. Conversely, we noticed some signals that were markedly more intense in case of the leaf samples compared to the trichome samples where these signals were either absent or at least strongly depleted (Fig. 6B). Taken together, the outcome of the qRT-PCR and SDS-PAGE analyses indicates an enrichment of trichome-specific RNA and detectable differences in the trichome and leaf proteomes.

### Mass spectrometric analysis of the *A. thaliana* trichome proteome

For a profound evaluation of the trichome proteome, we subjected four replicates of our RAW and PURE trichome samples, each purified from individual pools of seedlings grown in two independent batches, as well as the corresponding leaf reference samples (LEAF(+) and LEAF(−)) to nano-liquid chromatography coupled to tandem mass spectrometry (LC–MS/MS). By this label-free quantitative proteome analysis we identified 2,669 proteins, of which 2,240 were quantified in at least three replicates of one of the experimental conditions (Additional file 2: Data S1). In the comparison of PURE samples with LEAF(−) samples, 906 proteins showed significant differences in accumulation (Student´s *t*-test *q*-value < 0.05, corrected for multiple hypothesis testing with a permutation-based FDR < 0.05, and at least twofold change in abundance). We plotted the signal ratio between PURE and LEAF(-) samples *versus* the statistical significance of the difference to illustrate the enrichment or depletion of proteins in trichomes (Fig. 6C). Among the 2,240 proteins, 223 were significantly and at least two-fold enriched in the trichome PURE fraction compared to LEAF(−) samples whereas 683 proteins were depleted. We had a closer look at the identities of the top ten enriched and depleted proteins in trichomes after DGC upon comparison of PURE trichome samples with LEAF(−) reference samples (Fig. 6C). Whereas the top 10 trichome-depleted proteins are primarily associated with photosynthesis, the top 10 trichome-enriched proteins comprise various functions such as vesicle trafficking (Clathrin heavy chain 2), heavy metal transport (Copper transporter 5) and carbohydrate processing (probable polygalacturonase, probable pectinesterase/pectinesterase inhibitor 35, UDP-glycosyltransferase superfamily protein).

### Trichome-enriched proteins are involved in carbohydrate and secondary metabolism as well as vesicle trafficking

**Fig. 7:**
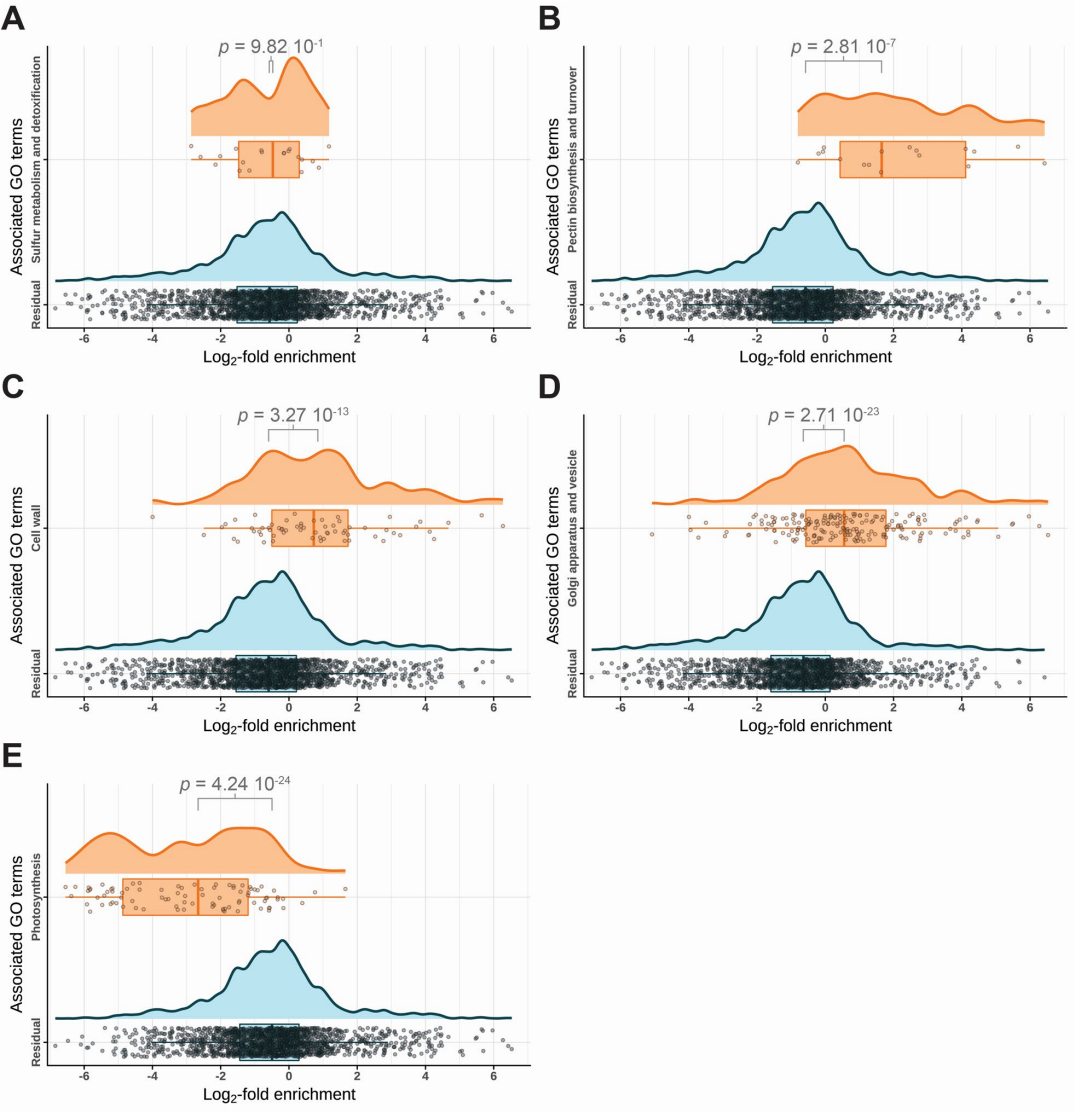
Functional groups of GO terms associated with proteins resident in *A. thaliana* trichome and/or leaf samples. Proteins identified by proteomic analysis were filtered for their association with groups of specific GO terms (orange) and compared to proteins not associated with these GO terms (Residual, blue) concerning fold enrichment in PURE samples compared to LEAF(−) samples. Dots represent proteins identified in at least three of four measured samples whereas box plots and curves indicate the probability distribution of these proteins regarding fold enrichment. **A** Sulfur metabolism and detoxification. **B** Pectin biosynthesis and turnover. **C** Cell wall. **D** Golgi apparatus and vesicle. **E** Photosynthesis. Statistical significance of the difference between GO term-associated and not-associated proteins is indicated by *p*-values above square brackets (Wilcoxon-Mann–Whitney test). Filter criteria for GO term groups are listed in Table S2

We further subjected trichome-enriched (PURE) and purification-depleted proteins (LEAF(−)) to gene ontology (GO) term enrichment analysis with PLAZA 4.5 [33]. For the PURE fraction, we found 60 associated GO terms that were at least two-fold enriched compared to all *A. thaliana* proteins (Additional file 1: Fig. S6). Likewise, we found 175 enriched GO terms associated with proteins depleted in trichomes of which the 75 most significantly enriched are shown in Additional file 1: Fig. S6. GO terms linked to trichome-enriched proteins were, amongst others, associated with vesicle trafficking and carbohydrate metabolism (Additional file 1: Fig. S6). Furthermore, we found that proteins probably involved in strictosidine synthesis were strongly overrepresented in trichomes, showing an enrichment of ~ 30 compared to the total *A. thaliana* proteome (Additional file 1: Fig. S6). For trichome-depleted proteins, we mainly found GO terms linked to photosynthesis (Additional file 1: Fig. S7). Apart from the *A. thaliana* proteome, we also used our identified 2,240 proteins as reference data to score the enrichment or depletion of selected groups of GO terms. For this purpose, we plotted the subset of proteins within our data associated with a particular group of GO terms *versus* the fold enrichment (compared to LEAF(−)) of those proteins and compared the very subset to all proteins that are not linked to this group of GO terms (Fig. 6, Additional file 1: Table S2). In contrast to an earlier study [20], we found that proteins associated with sulfur metabolism and detoxification were not particularly strongly enriched in our trichome preparations (Fig. 7A). On the contrary, proteins related to pectin synthesis and turnover, the cell wall or the Golgi apparatus and vesicle trafficking were enriched in trichome samples compared to leaf samples (Fig. 7B–D). In good agreement with what was indicated by GO term comparison with the whole *A. thaliana* proteome, proteins linked to photosynthesis were depleted in trichome samples within our data set (Fig. 7E).

## Discussion

During the first decade of the new millennium, various strategies for trichome isolation were proposed [20, 22, 25, 26], probably reflecting the increasing relevance of trichomes as a model for cell development and differentiation [4]. The most recent of these methods was published by Marks and co-workers and rests upon trichome release by the shared impact of EGTA and a physical stimulus [25]. Though this method is superior compared to previously introduced trichome isolation strategies in terms of experimental effort and trichome yield, we identified space for further improvement. By addressing certain drawbacks such as trichome shearing during release, scalability and inconvenient trichome purification by forceps, we aimed to take the innovative EGTA-based trichome release to the next level.

We found that an alkaline buffer without EGTA (ROLLER) reduces the trichome yield by 99% compared to the original method (VORTEX) (Fig. 2A, B), underlining the necessity of EGTA for trichome release. EGTA chelates bivalent cations, and during trichome release it is thought to weaken the connection between trichomes and the leaf surface by capturing calcium ions, which are mandatory for the cross-linking of the pectic matrix. Because of its larger size, EGTA is commonly preferred over other chelating agents like EDTA to capture calcium ions. However, EGTA, a pricy chemical, is the main cost driver of the trichome isolation process and, depending on the supplier, can be more than 50-times more expensive than EDTA. Our data show that EGTA can be substituted by EDTA (Additional file 1: Fig. S1), probably because of the excess of chelating agents in the release buffer (50 mM) compared to typical extracellular calcium concentrations, which are in the low millimolar range [34, 35]. In addition, we compared the original method relying on high-speed short-term mixing (VORTEX) to a gentler low-speed long-term stirring strategy (STIRRER). We found that the latter so-called STIRRER method yields about 62% more trichomes than the original VORTEX method, yet showing similar reproducibility between experimental replicates (Fig. 2A, B). Because the STIRRER method relies on beakers and not on single-use reaction tubes as for the VORTEX method, it furthermore reduces disposable waste as well as process costs and enables process scale-up to, for instance, bioreactor size. The latter might be a first step to utilize trichomes for biotechnological applications like the production of secondary metabolites, the reduction of host cell proteins during recombinant protein production, or the harvest of cellulose-rich plant fibers like cotton seed trichomes [36, 37].

In this context, we showed that the STIRRER method is suitable to release trichomes from leaves of plant species other than *A. thaliana* (Fig. 3). As we found considerable differences in the trichome mass recovered from *S. lycopersicum*, *S. tuberosum*, *N. benthamiana* and *H. annuus.* (Additional file 1: Table S1), we assume that our method requires a certain degree of streamlining according to the respective species. For instance, the majority of trichomes isolated from *H. annuus* was recovered during the third cycle of filtration (see Methods section), indicating that incubation time in EDTA-PBS buffer requires prolongation. Furthermore, in contrast to *A. thaliana*, all of the tested species possess multiple types of glandular and/or non-glandular trichomes [38–41]. Accordingly, the appropriate incubation time for trichome release probably also differs depending on trichome type. For example, microscopy of trichomes isolated from *H. annuus* revealed that almost exclusively non-glandular trichomes were recovered (Fig. 3, RAW) whereas trichomes of the linear glandular type [42] were largely retained on the adaxial epidermis (Fig. 3, LEAF(−)). Likewise, we identified primarily non-glandular trichomes of the types II and V [39] in our *S.* *tuberosum* RAW samples (Fig. 3, RAW). For the closely related *S. lycopersicum*, we found at least four different of the overall seven described trichome types (Additional file 1: Fig. S2A) [38, 43]. According to our assessment, these trichomes represent the glandular type I and type VI trichomes as well as non-glandular trichomes of the types II and III [44]. In case of *N. benthamiana*, microscopy revealed two types of glandular trichomes (Additional file 1: Fig. S2B). As, to the best of our knowledge, trichome morphology was only published for *N. tabacum* to date [41], we decided to stick with the proposed terminology and refer to *N. benthamiana* trichomes as tall and short. Our micrographs show that we isolated an immense amount of short glandular trichomes from *N. benthamiana* whereas tall trichomes were scarce (Additional file 1: Fig. S2B). Micrographs of our respective LEAF(+) samples suggest that these relations are an actual representation of the trichome ratios on the plants rather than an artefact of the isolation procedure (Fig. 3, LEAF( +)). Strikingly, tall trichomes of *N. benthamiana* depicted a characteristic dark coloration of the second and occasionally third stalk cell. In contrast to *A. thaliana*, all aforementioned plant species harbor glandular trichomes that are known for their ability to produce and secrete secondary metabolites. Apart from their natural purpose in plant defense, some of these metabolites became commercially relevant as they potentially function as essential oils, drugs or pesticides [45–47]. Hence, it is not surprising that publications about glandular trichomes skyrocketed in the last decade, especially those related to -omics biology [48]. In this context, glandular trichomes of *S. lycopersicum* were studied intensively in the past [43, 49], probably sparked by rich genetic resources and extensive sequence data available for this species [50]. For instance, *S. lycopersicum* type VI trichomes were subjected to transcriptomics, proteomics, metabolomics and ^13^C-labelling in a single study [51] after trichome isolation with the aid of a frozen paint brush [52]. Though -omics analyses of trichomes were also carried out for plants like *H. annuus* and members of the genus *Nicotiana* [40–42, 53–56], the amount of data is scarce compared to *S. lycopersicum* and closely related species. Additionally, most of the available studies focus on only a single, often highly abundant trichome type and lack comprehensive comparisons between different glandular and non-glandular trichomes. We are aware that our data provide only a superficial view on the versatile nature of trichomes in plants apart from *A. thaliana*. Nonetheless, we hope that the demonstration that EDTA/EGTA-mediated trichome release is suitable for glandular and non-glandular trichomes of different plant species will be helpful to address some compelling open questions, especially concerning the biology of glandular trichomes [48].

For any biotechnological processing or certain (-omics) analyses carried out in basic research, an efficient strategy to enhance trichome purity after release and enrichment is desirable. In our hands, the burden of leaf-specific impurities in the samples after trichome release was high (Fig. 1), even after removal of crude debris by forceps as suggested by Marks and colleagues [25]. Thus, we added a DGC step after trichome enrichment, which considerably reduced greenish appearance and crude foliaceous plant debris in our samples (Fig. 2C). Purification of trichomes by DGC benefits from the high density of trichomes, which passed the 80% layer of our sucrose density gradient whereas regular leaf tissue remnants congregated either at the interface of the 40%/80% or the 20%/40% layers. Furthermore, the sedimentation of trichomes during DGC facilitated the removal of fractions for disposal and subsequent washing of the enriched trichomes. Trichome quantification revealed that DGC does not cause a significant reduction in trichome yield (Fig. 2A). However, we observed that prolonged stirring times (> 1 h) enhanced the number of trichomes that do not pellet during DGC. We speculate that this decrease in trichome density might be due to a sluice of cytosolic trichome content during stirring. We, thus, recommend starting trichome enrichment after 30 min and then proceed in a circulatory system as described in the Methods section. This might be of relevance for downstream analyses that particularly focus on cytosolic material such as transcriptomics, proteomics or metabolomics.

As a first downstream analysis step, we applied histochemical stains to our isolated trichomes. Comparing trichomes released by the STIRRER or VORTEX method as well as purified and unpurified trichomes, we detected no difference regarding histochemical staining patterns for callose (Aniline Blue), cellulose (Calcofluor White), and lipids (Sudan Black B) (Fig. 4). However, trichomes released by the VORTEX method were stained considerably better after 5 min of incubation in a Toluidine Blue O solution than trichomes released by the STIRRER method (Fig. 4A). We speculate that this increased staining efficiency of Toluidine Blue O might be caused by (i) a better accessibility of the trichomes for the dye or, probably less likely, (ii) an increased level of polygalacturonic acid that reacts with Toluidine Blue O to a pinkish purple molecule [31]. Both, better dye accessibility and higher polygalacturonic acid content might be conditioned by the harsh mixing conditions during VORTEX release, which may cause trichome shearing leading to injuries and/or degradation of pectic polysaccharides. Adding to this, we found that trichomes released by the STIRRER method are stained after prolonged incubation times (> 1 h) in Toluidine Blue O solution (Additional file 1: Fig. S3A), indicating that the lack of staining after 5 min of incubation time is not due to a sluice of pectic polysaccharides during STIRRER release. Upon Ruthenium Red staining, we identified reddish congregations in our unpurified samples sticking to the trichome surface. Those impurities, which are probably leaf debris with a high content of pectic polysaccharides, were reduced after DGC purification. Regardless of these method-related differences in trichome staining, we found that both VORTEX- and STIRRER-derived samples as well as DGC-purified and non-purified trichomes are suitable to carry out histochemical analyses of trichome cell walls. For instance, we probed the previously described Ortmannian Ring [18], located at the interface between basal and apical trichome region, by Aniline Blue in all samples. We also observed the Ortmannian Ring after Calcofluor White and Toluidine Blue O staining as well as Mäule reaction (Fig. 4, Additional file 1: Fig. S3B), indicating that the Ortmannian Ring is rather a complex congregation of multiple cell wall components than a simple callose-rich region within the trichome.

After histochemistry, we quantified the content of neutral matrix monosaccharides and crystalline cellulose in trichomes isolated by either the VORTEX or the STIRRER method. Within this analysis, we also tested Nycodenz as an alternative density gradient medium because the sucrose used during DGC potentially distorts values obtained for matrix polysaccharides and cellulosic glucose. Though we found no considerable differences between glucose and cellulose contents regarding the purification strategy (Fig. 5A, D), we strongly recommend either extensive washing of the trichomes after purification by sucrose DGC or the use of Nycodenz for gradient assembly. Regarding differences related to the release strategy, we observed a higher glucose content in trichomes processed by the VORTEX method compared to trichomes processed by the STIRRER method, indicating a reduced hemicellulose content in the latter. This conclusion, however, remains doubtful because other monosaccharides found within hemicelluloses such as xylose and mannose showed no difference irrespective of the release strategy (Fig. 5A). For monosaccharides associated primarily with pectic polysaccharides and/or arabinoglactan-proteins including rhamnose, arabinose and galactose, we found increased CoVs among the VORTEX samples compared to the STIRRER samples (Fig. 5C). This increased intra-method variation underpins our assumption that harsh mixing conditions during trichome release by the VORTEX method may interfere with the integrity of the pectic and/or arabinoglactan-protein matrix. Upon comparison of our data for trichome wall monosaccharides with those published by Marks and co-workers [25], we noticed a fairly similar distribution in terms of monosaccharide proportions within the two data sets. Solely the values for xylose and mannose appear to be shifted (Additional file 1: Table S3). The higher xylose/mannose ratio in our trichomes might be a consequence of the age difference between our *A. thaliana* plants (~ 42 d) and those used by Marks and colleagues [25] (~ 28 d). Former studies support this assumption as they show that the xylose/mannose ratio seems to be proportional to the degree of lignification [57] while the latter is an inherent characteristic of secondary and thus mature cell walls. Here, we want to point out that the proportion of mature trichomes is a matter of leaf size, as fresh trichomes develop at the periphery of the leaf blades [5, 58]. Leaf size, however, is age-dependent and, thus, older plants may be preferred for the investigation of mature trichomes. A previous study estimated the crystalline cellulose content of *A. thaliana* trichomes to be approx. 30% [59], similar to our results here (Fig. 5D). Thus, despite being modified epidermal cells, trichomes may be rather comparable to stem tissue in terms of cellulose content than to leaf or seedling tissue (Additional file 1: Table S3).

We performed SDS-PAGE with total protein extracts and found considerable differences in the signal patterns between leaf and trichome samples after gel staining (Fig. 6B). The depletion of some proteins in the trichome samples likely reflects a reduction of, for instance, abundant photosynthesis-related proteins like ribulose-1,5-bisphosphate carboxylase-oxygenase (RuBisCO) large (~ 53 kDa) or small subunit (~ 20 kDa). Conversely, we observed signals that were less pronounced or absent in the leaf samples, likely representing proteins that are more abundant in trichomes than other leaf cell types. We aimed to unravel the identities of these differentially present proteins by subjecting trichome and leaf protein extracts to LC–MS/MS, which yielded a set of 223 trichome-enriched proteins and confirmed the depletion of proteins linked to photosynthesis in the trichome samples (Fig. 6C). This finding is in good agreement with the prevailing opinion that trichomes are not photosynthetically active in *A. thaliana* [60, 61].

The only other *A. thaliana* trichome proteome published yet comprised a set of 63 proteins [20] including a striking number of proteins involved in sulfur metabolism and detoxification. Like Wienkoop and co-workers, we also identified methionine synthases (At5g17920, At3g17390, At1g02500, At4g39460, glutaredoxins (At2g36880, At5g40370) and other glutathione-associated proteins (At4g29210, At1g69820, At1g69820), but could not confirm their strong enrichment in trichomes compared to the residual leaf proteome (Fig. 7A). One potential explanation for this apparent discrepancy is that Wienkoop and colleagues did not analyze their trichome proteome in relation to seedling or leaf reference samples. Therefore, we suppose that though indeed abundant in trichomes, sulfur- and/or detoxification-linked proteins are not an exclusive characteristic of trichomes. However, we cannot exclude that the difference also arises from discrepancies in the investigated plant material or applied sampling methodology, as Wienkoop et al*.* mechanically harvested trichomes from *A. thaliana* leaves frozen in liquid nitrogen whereas our harvesting strategy involved prolonged submersion of the seedlings in liquid media at room temperature. Apart from that, a probable pectinesterase (At2g43050), which ranked at position nine in the Wienkoop proteome [20], was the second most enriched protein in trichomes among our 2240 identified proteins (Fig. 6C, Additional file 2: Data S1). Transcript accumulation of the respective gene was also trichome-enriched in the transcriptomes published by Marks et al. [27] and Jakoby et al. [32].

Our set of 223 trichome-enriched proteins lists several proteins linked to wall pectin biosynthesis and turnover, including pectin esterases (At2g43050, At3g43270) and acetylesterases (At5g45280), polygalacturonases (At1g80170, At4g23820) and trichome birefringence-like 38 (At1g29050), a putative pectin O-acetyltransferase (Fig. 7B, Additional file 2: Data S1). In particular, pectin O-acetyltransferases are known to impact cellulose deposition in trichomes [59, 62]. The presence of enriched GO terms associated with the cell wall such as “cell wall modification”, “cell wall organization” and “plant-type cell wall” (Fig. 7C) suggest that trichomes are subject to extensive processing of their cell wall matrix. Adding to this, pectins and hemicelluloses are synthesized in the Golgi apparatus [63, 64], which is in good agreement with the enrichment of GO terms linked to this organelle and vesicle trafficking (Additional file 1: Fig. S6, Fig. 7D). Eventually, we found strictosidine synthase-like (SSL) proteins (SSL12: At1g74020, SSL3: At1g08470, SSL9: At3g57020) among our enriched trichome proteins (Fig. 6C, Additional file 2: Data S1), as well as a strong enrichment of the respective related GO term (Additional file 1: Fig. S6). Though none of these SSLs was discovered by the Wienkoop proteome [20], *SSL* transcripts were found to be enriched in the published trichome transcriptomes [27, 32]. Strictosidine synthases catalyze the formation of precursors during the biosynthesis of monoterpenoid indole alkaloids, though it is still elusive if SSLs also take over this function in *A. thaliana* and whether the model plant can in fact biosynthesize complex alkaloids [65, 66]. Some SSLs like SSL4–7 are suspected to be involved in plant defense in *A. thaliana* [67]. This notion is further supported by the fact that *SSL9* expression seems to be jasmonic acid-dependent [68]. Collectively, the data suggest that trichome-localized SSL-derived secondary metabolites may play a yet unrecognized role in plant defense. This would be in line with the widely accepted function of non-glandular trichomes as protective structures that shield plant organs from various abiotic and biotic stresses [69].

Apart from proteomic analyses of glandular trichomes in various species such as Cannabis (*Cannabis sativa*; [70]), tomato (*Solanum lycopersicum*; [71]), hop (*Humulus lupulus*; [72]) and *Artemisia annua* [73], to the best of our knowledge only one further analysis of non-glandular trichomes (in olive; [74]) has been published. Since glandular trichomes are highly specialized for the biosynthesis and secretion of dedicated, taxon-specific secondary metabolites, the comparison of their proteomes with our proteomic data derived from non-glandular *A. thaliana* leaf trichomes is of limited value. Analysis of the proteome of non-glandular peltate trichomes of olive leaves revealed, however, the presence of a number of enzymes involved in abiotic and biotic stress responses. In good agreement with our data, Roka and colleagues found a trichome birefringence-like protein and an endo-1,3-β-glucosidase to be overrepresented in olive trichomes. The latter represents a homolog of *A. thaliana* pathogenesis-related 2 (At3g57240), which is a member of the Glycoside Hydrolase 17 family (endo-1,3-β-glucosidases) and also abundant in trichomes according to our data (Additional file 2: Data S1). Strikingly, we identified four additional endo-1,3-β-glucosidases (At3g07320, At5g42720, At4g26830, At3g55430) that were highly enriched in trichomes (Additional file 2: Data S1), further reinforcing the presumed role of trichomes as general structural and chemical stress barriers [74].

## Conclusions

We present an advanced procedure for the isolation and purification of *A. thaliana* trichomes, which according to our data can be also adopted to other plant systems. We demonstrate that the yield, purity and quality of the isolated trichomes is sufficient for various types of downstream analyses, including histochemical staining, biochemical assays, as well as transcriptomic and proteomic analyses. We employed the procedure to perform proteomic analysis of *A. thaliana* trichomes, which enabled us to obtain a comprehensive reference data set of trichome-resident and -enriched proteins. This detailed insight into the trichome proteome promises to advance our understanding of trichome biology. The high yield and quality of the trichomes enriched by our procedure might be particularly advantageous for assays in which the quantity, integrity and purity of the sample material is critical.

## Methods

### Plant cultivation and harvest

*A. thaliana* Col-0 wild-type plants were sown on SoMi 531 soil (HAWITA, Vechta, Germany) in a propagation box with sieve bottom (500 × 320 × 60 mm, Wiesauplast, Wiesau, Germany) that was overlaid with a perforated metal plate (470 × 270 × 1 mm), similarly as described before [25]. Subsequently, the propagation box was placed in a large propagation tray (600 × 400 × 60 mm) for irrigation. The plants were cultivated at 22/20 °C average day/night temperature with a photoperiod of 10 h d^−1^, a photosynthetic photon flux density of 80–100 µmol m^−2^ s^−1^ and a relative humidity of 80–90%. At 42 d after sowing, the seedlings were harvested using a razor blade and thoroughly washed with tap water as described before [25]. For the isolation of trichomes from plants other than *A. thaliana*, young leaves of *S. lycopersicum*, *S. tuberosum*, *N. benthamiana* and *H. annuus* were harvested at 163 d, 43 d, 56 d or 43 d after sowing, respectively. *S. lycopersicum*, *S. tuberosum* and *H. annuus* were cultivated in a greenhouse with a photoperiod of 16 h d^−1^, whereas *N. benthamiana* was cultivated as described for *A. thaliana*.

### Trichome release by speed mixing (VORTEX)

About 36 g of freshly harvested *A. thaliana* seedlings were evenly distributed among eight 50-mL reaction tubes each containing 100 mg of glass beads (250–500 µm diameter, Carl Roth, Karlsruhe, Germany) and 15 mL of a solution containing 50 mM EGTA or EDTA (Carl Roth, Karlsruhe, Germany), respectively (pH 8.0, adjusted with KOH, Sigma-Aldrich, St. Louis, USA), in phosphate-buffered saline (PBS). The PBS solution was prepared as described before [75]. The tubes containing the *A. thaliana* seedlings were mixed in three cycles for 30 s with a 30 s pause between each cycle using a Vortex-Genie 2 (Scientific Industries, Bohemia, USA) reaction tube mixer. It was important to prevent overloading of the tubes with the seedlings to ensure free rotation of the latter during mixing.

### Trichome release by magnetic stirring (STIRRING)

About 36 g of freshly harvested *A. thaliana* seedlings were transferred to a 500-mL beaker containing 1000 mg of glass beads (250–500 µm diameter) and 240 mL of a PBS solution containing 50 mM EGTA or EDTA, respectively. Note that for the isolation of trichomes from plants other than *A. thaliana*, 15 g leaf material was processed and exclusively EDTA-PBS buffer was used. The trichome buffer suspension was stirred using an IKAMAG Combimag RCT magnetic stirrer (IKA, Staufen, Germany) at 300 rpm for 30 min with a triangular stirring bar of 80 mm in length. After stirring, the suspension was used for trichome enrichment as described below. Following a first round of agitation and filtration, the process was repeated two times for 15 min each. For this purpose, the EGTA/EDTA-containing PBS buffer was captured during cell strainer filtration and reused.

### Trichome release by alkaline buffer (ROLLER)

About 100 g of freshly harvested *A. thaliana* seedlings were incubated for 24 h in a destaining solution (3:1 ethanol-(techn. 96.0% (v/v)) acetic acid (100% (v/v))). Subsequently, about 12.5 g of the seedlings were transferred to a single 50-mL reaction tube containing 30 mL di-potassium hydrogen phosphate buffer (0.1 M, pH 9.5, adjusted with 10 M KOH) buffer and 100 mg of glass beads (250–500 µm diameter). Overall, eight 50 mL tubes were incubated on a RS-TR 6/10 tube roller (PHOENIX Instrument, Garbsen, Germany) for 24 h.

### Trichome enrichment

Four layers of screen door mesh were used to separate the processed seedlings and the buffer solution containing the released trichomes. The latter was captured in a 300 mL beaker. During the filtration process, the seedlings were rinsed with PBS to free trichomes trapped in-between the residual plant material. The trichome-PBS suspension was poured through a cell strainer (VWR, Radnor, USA) with a nominal pore size of 100 µm. Clogging of the cell strainer was prevented by allowing the trichomes to settle at the bottom of the beaker prior to filtration. The restrained trichomes were collected by a small spatula and transferred to 2-mL reaction tubes. Enriched trichomes were stored at 4 °C in-between the enrichment cycles. After enrichment, trichome fresh mass was measured and trichomes were evenly distributed among two 2-mL reaction tubes. Whereas one of these tubes was frozen in liquid nitrogen and stored at − 80 °C for further experiments, trichome purity in the remaining sample was increased by DGC (see below).

### Trichome purification

Remaining plant debris in the trichome samples was reduced by applying the trichome suspension to a discontinuous sucrose/Nycodenz (Progen, Heidelberg, Germany) gradient for DGC. For this purpose, 1 mL of the trichome suspension was applied to a sucrose/Nycodenz gradient consisting of four layers with different sucrose (0%, 20%, 40%, 80% (m/v)) or Nycodenz (0%, 15%, 30%, 60% (m/v)) concentrations. Gradient layering was carried out using a syringe and a canula in a 15-mL tube by applying 1 mL of the lowest-density layer first and then carefully layering the layer with the next higher density below. After layering, the suspension with the purified trichomes was applied carefully (cut pipette tip) to the density gradient and the 15-mL tubes were centrifuged in a swing-out rotor at 3000*g* for 10 min at room temperature. Subsequently, the layers were carefully separated (cut pipette tip) and transferred to 15-mL reaction tubes. These tubes were filled with PBS, mixed and centrifuged at 3000*g* for 3 min at room temperature. Afterwards, the supernatant was discarded and washing with PBS repeated. Eventually, the trichome samples were stored either in 1 mL PBS at 4 °C for immediate use or at − 80 °C after removal of excess liquid by a pipette or lyophilization using an Alpha 1–2 LDplus freeze dryer (Martin Christ, Osterode am Harz, Germany).

### Determination of trichome yield

Trichome yield was determined by microscopic quantification. For this purpose, the samples were mixed thoroughly and subsequently 20 µL of the trichome suspension was transferred to microscopy slides (cut pipette tip). All trichomes of the sample were counted. Subsequently, the number of trichomes per seedling fresh mass was calculated as described below (Eq. 1).

1 $${Tr}_{FM}= \frac{Tr \cdot 50}{FM \cdot \frac{1}{2}}= \frac{Tr \cdot 100}{FM}$$

Equation 1: Calculation of the trichome number per seedling fresh mass per mL resting upon quantification and extrapolation of a 20 µL sample. Tr_FM_: trichome number per fresh mass, Tr: trichome number, FM: fresh mass.

### Histochemical staining of isolated trichomes

Histochemical staining of *A. thaliana* trichomes was carried out using 20 µL of the respective trichome suspension sample, which was mixed to equal shares with a desired staining solution. Incubation took place in the dark. Incubation times and microscopic illumination procedures are given for each dye in Additional file 1: Table S4. Calcofluor White, Ruthenium Red, Toluidine Blue O and Mäule staining solutions were prepared as described by Mitra and Loqué [31]. For Aniline Blue staining, 0.01% (m/v) Aniline Blue was dissolved in 150 mM dipotassium hydrogen phosphate buffer. Sudan Black B staining solution was prepared by dissolving 0.001% (m/v) Sudan Black B in 70% (v/v) ethanol. Deviating from the before described procedure, Sudan Black B staining solution was soaked between the cover glass and the microscopy slide carrying the trichomes to avoid the contamination of the sample with Sudan Black B precipitates. Micrographs were captured using a BZ-9000 transmitted light and fluorescence microscope (Keyence, Osaka, Japan). Image optimization was carried out using Adobe Photoshop 2021 in an identical manner for all micrographs of the same staining type.

### Quantification of neutral sugars and cellulose in cell walls of *A. thaliana* trichomes

The mass of neutral monosaccharides and cellulose in cell walls of isolated and lyophilized trichomes was determined as described previously [76]. In brief, alcohol insoluble material was prepared from the dried trichomes and split into two samples—one sample was treated with a weak acid to release matrix polysaccharide-derived sugars, while the other sample was treated with a strong acid to swell cellulose followed by a weak acid to yield monosaccharides derived from cellulose and the matrix polymers. Subtraction of the two values allows for the quantification of cellulose. Monosaccharides were measured by high-performance anion-exchange chromatography with a pulsed amperometric detector (HPAEC-PAD). Quantification of cell wall monosaccharides and cellulose was carried out once for each condition.

### RNA and protein extraction

After the addition of glass beads (0.75–1.00 mm diameter, Carl Roth, Karlsruhe, Germany), lyophilized trichomes (~ 10 mg dry mass) or leaf material (two leaves) were frozen in liquid nitrogen and ground three times for 1 min at 30 s^−1^ using a bead mill (Retsch, Haan, Germany). Between each grinding step, the samples were re-frozen in liquid nitrogen. RNA was extracted according to US Patent No. 5,973,137 and described in [77]. Departing from the latter, the tissue powder was suspended in 600 µL lysis buffer (2% (m/v) SDS, 68 mM sodium citrate, 132 mM citric acid, 10 mM EDTA) and subsequently, 200 µL protein precipitation buffer (4 M sodium chloride, 17 mM sodium citrate, 33 mM citric acid, pH 3.5) was added. RNA was stored at − 80 °C. Furthermore, 500 µL isopropanol and ethanol (70% (v/v)) volume were used for precipitation or washing, respectively. For protein extraction, tissue powder was thoroughly suspended in 100 µL protein extraction buffer (2.5 mM EDTA, 100 mM HEPES, 4% SDS) using a pipette and vacuum infiltration was carried out for 5 min. After centrifugation at 10,000*g* for 1 min at room temperature, the supernatant was transferred to a fresh 1.5 mL reaction tube and the protein extracts were stored at − 20 °C.

### DNase digest and reverse transcription

Genomic DNA was removed from trichome and leaf RNA samples by mixing 0.5 µL DNase I (Thermo Fisher Scientific, Waltham, USA) with 1 µL DNase I buffer and 9 µL RNA solution (500 ng). The mixture was incubated for 15 min at 25 °C and subsequently the reaction was stopped by adding 0.5 µL 50 mM EDTA solution and heating to 75 °C for 10 min. Reverse transcription of RNA to cDNA was carried out using SuperScript II reverse transcriptase (Thermo Fisher Scientific, Waltham, USA) according to the instructions provided by the manufacturer. cDNA samples were stored at − 20 °C.

### qRT-PCR and validation of amplicon integrity

Primers for qRT-PCR were designed using Clone Manager 9 Professional Edition (Sci-Ed, Westminster, USA). Among others, constraints for primer selection were primer length (18–22 bp), similar melting temperatures (58–65 °C), GC content (40–60%) and amplicon size (100–200 bp). The selected primers were synthesized by IDT (Coralville, USA) and are listed in Additional file 1: Table S5. Samples for qRT-PCR were assembled using the Takyon No ROX SYBR® MasterMix blue dTTP (Eurogentec, Seraing, Belgium) in a total reaction volume of 5 µL (2.5 µL Takyon MasterMix, 0.125 µL forward/reverse primer (10 µM), 2 µL cDNA (1:10), 0.5 µL nuclease-free water). Subsequently, the LightCycler® 480 II system (Roche, Basel, Switzerland) was used according to the instructions for three-step cycles (annealing: 58 °C) provided by the master mix manufacturer. Fold-change of *AtEXO70H4* expression compared to LEAF(−) samples was computed by the 2^−∆∆CT^ method using *AtACT2* and *AtTUB4* as reference genes [78]. After qRT-PCR, amplicons were separated by agarose gel electrophoresis (1% (m/v) agarose in TAE buffer, 100 V, 40 min) and detected using a GelDoc™ XR + (Bio-Rad, Hercules, USA) device. Furthermore, amplicons were purified prior to sequencing by the NucleoSpin Gel and PCR Clean-Up kit (Macherey–Nagel, Düren, Germany). Sanger sequencing was carried at Eurofins (Luxembourg) using the primers listed in Additional file 1: Table S5 and alignments were computed in Clone Manager 9.

### SDS-PAGE and SYPRO Ruby staining

Due to the high burden of SDS in our protein extracts, protein concentrations in trichome and leaf extracts were determined by the detergent-resistant Pierce BCA protein assay (Thermo Fisher Scientific, Waltham, USA). Subsequently, 7.5 µg protein were prepared for separation per SDS-PAGE by adding NuPAGE LDS sample buffer (4×) as well as NuPAGE reducing agent (10×) (Thermo Fisher, Waltham, USA) and incubation at 95 °C for 10 min for denaturation. Samples were loaded on Bis–Tris gels (4% stacking gel, 12% resolving gel) and gels were run at 175 V for 60 min in MES buffer. SYPRO Ruby gel staining (Thermo Fisher Scientific, Waltham, USA) was carried out according to the instructions provided by the manufacturer and signals were detected using a ChemiDoc™ XRS + (Bio-Rad, Hercules, USA) device.

### Proteome analysis

For proteome analysis, four replicates per sample type (LEAF(+), LEAF(−), RAW, PURE), originating from four independent trichome isolation experiments in two independent seedling batches, were analyzed by LC–MS/MS. For each sample, 20 µg protein extract in SDS-buffer was denaturated at 60 °C for 5 min, followed by reduction with 10 mM (final concnetration) dithiothreitol (DTT) at 37 °C under constant shaking (700 rpm) for 30 min and cysteine alkylation with 50 mM chloroacetamide (final concentration) in the darkness for 30 min. The reaction was quenched by adjustment to a final concentration of 50 mM dithiotreitol (DTT) at room temperature for 30 min. Proteins were purified using single-pot solid-phase (Sp3) beads [79], resuspended in 100 mM HEPES, 2.5 mM CaCl_2_, pH 7.5, and digested at a protein:enzyme ratio of 100:1 by addition of 0.2 µg typsin (SERVA, Heidelberg, Germany) at 37 °C under constant shaking (700 rpm) overnight. The reaction was stopped by acidification to pH < 3 with formic acid and peptides desalted using self-prepared double-layer C18 STAGE-tips [80].

An estimated 1 µg of desalted peptide samples were analysed using an Ultimate 3000 RSLCnano chromatography system (Thermo, Bremen, Germany), equipped with a C18 trapping column and a 50 cm µPAC C18 analytical column (both PharmaFluidics, Ghent, Belgium), connected to an Impact II high resolution Q-TOF mass spectrometer (Bruker, Bremen, Germany) via a CaptiveSpray nano ESI source (Bruker, Bremen, Germany) as described previously [81].

Peptides were identified by matching acquired tandem mass spectra to protein entries in the UniProt *Arabidopsis* protein database (date of download 2021-02 with appended potential contaminants and a reverse-decoy database) using the MaxQuant software suite [82] v2.0.1 with standard settings for Bruker QTOF instruments, with the exception of minimum MS1 intensity, which was set to 30. Trypsin was set as digestion enzyme (specific), carbamidomethylation was considered as fixed cysteine modification and N-terminal acetylation and methionine oxidation were considered as variable modifications. The function “match between runs” was enabled only within each of the four groups due to the strongly divergent composition of the proteomes, and LFQ values were calculated using standard settings. A FDR of 0.01 was applied at the levels of peptide-spectrum matching and protein identification.

### Computation and statistical procedures

R v.4.1.0 (R foundation, www.r-project.org/) was used for plotting, statistical analyses and filtering of data during this study. Plotting was carried out using the ggplot2 library whereas filtering of proteome data was facilitated by the dplyr library both included in the tidyverse package [83]. Due to the nature of our data (normal distribution, equal variance, small sample size, unpaired groups), Student’s *t*-test with FDR correction was used to assess differences in trichome count for trichome RAW fractions, whereas paired *t*-test was used to compute *p*-values concerning differences in trichome numbers between RAW and PURE trichome fractions (paired groups). CoVs were calculated by dividing the standard deviation by the mean of experimental replicates.

For proteome data analysis, MaxQuant search results were further analyzed using Perseus v1.6.14. [84]. Reverse hits and potential contaminants were removed, datasets filtered for protein entries identified at least three times in at least one of the four sample groups, and average fold-changes in abundance calculated and tested for statistical significance by pairwise Student’s *t*-test (two-sample test, minimum valid values = 3 in one group, permuation-based FDR < 0.05). The web-based platform PLAZA 4.5 was used for GO term enrichment analysis [33]. Significance levels for GO term enrichment were predefined resting upon the size of the data set (trichome-enriched: 0.005, trichome-depleted: 0.001). For conciseness, plots include only those GO terms with a hierarchical rank of one and only the 75 most significantly enriched GO terms are shown for trichome-depleted proteins. Computation of *p-*values for the comparison of proteins associated or not-associated with particular GO terms was carried out by Wilcoxon-Mann–Whitney test.

## Supplementary Information

**Additional file 1: Figure S1.** Comparison of EGTA and EDTA as chelating agents for trichome release. **Figure S2.** Trichome types in RAW samples of *S. lycopersicum* and *N. benthamiana*. **Figure S3.** Microscopic evaluation of histochemical staining of *A. thaliana* wild-type trichomes with Toluidine Blue O or by Mäule reaction. **Figure S4.** Comparison of the densities of sucrose and Nycodenz for DGC. **Figure S5.** Verification of amplicon sequence integrity after qRT-PCR. **Figure S6.** GO terms associated with proteins enriched in trichomes. **Figure S7.** GO terms associated with proteins depleted in trichomes. **Table S1.** Trichome yield per harvested plant fresh mass. **Table S2.** GO terms and key words used as filter criteria to identify proteins of similar function among trichome and/or leaf samples. **Table S3.** *A. thaliana* neutral monosaccharide and cellulose amounts measured in various studies. **Table S4.** Stains, incubation times and illumination procedures used to probe cell wall components and lipids of trichomes. **Table S5.** Primers used in this study.

**Additional file 2: Data S1.** Overview of the proteomics data.

## Data Availability

The mass spectrometry proteomics data have been deposited to the ProteomeXchange Consortium via the PRIDE [85] partner repository with the dataset identifier PXD028808.

## Abbreviations

CoV: Coefficient of variation
DGC: Density gradient centrifugation
EDTA: Ethylene diamine *N*,*N*,*N*′,*N*′-tetraacetic acid
EGTA: Ethylene glycol-bis(β-aminoethyl ether)-*N*,*N*,*N*′,*N*′-tetraacetic acid
FDR: False discovery rate
GO: Gene ontology
PBS: Phosphate-buffered saline
qRT-PCR: Quantitative reverse transcriptase-polymerase chain reaction
SSL: Strictosidine synthase-like

## References

[1] Johnson HB (1975). Plant pubescence: an ecological perspective. Bot Rev.

[2] Suo B, Seifert S, Kirik V (2013). *Arabidopsis GLASSY HAIR* genes promote trichome papillae development. J Exp Bot.

[3] Schellmann S, Hülskamp M (2005). Epidermal differentiation: Trichomes in *Arabidopsis* as a model system. Int J Dev Biol.

[4] Hülskamp M (2004). Plant trichomes: a model for cell differentiation. Nat Rev Mol Cell Biol.

[5] Hülskamp M, Miséra S, Jürgens G (1994). Genetic dissection of trichome cell development in Arabidopsis. Cell.

[6] Larkin JC, Oppenheimer DG, Lloyd AM, Paparozzi ET, Marks MD (1994). Roles of the *GLABROUS1* and *TRANSPARENT TESTA GLABRA* genes in Arabidopsis trichome development. Plant Cell.

[7] Rerie WG, Feldmann KA, Marks MD (1994). The *GLABRA2* gene encodes a homeo domain protein required for normal trichome development in *Arabidopsis*. Genes Dev.

[8] Koornneeff M, Dellaert LW, van der Veen JH (1982). EMS-and relation-induced mutation frequencies at individual loci in *Arabidopsis thaliana* (L.) Heynh. Mutat Res..

[9] Payne CT, Zhang F, Lloyd AM (2000). *GL3* encodes a bHLH protein that regulates trichome development in Arabidopsis through interaction with GL1 and TTG1. Genetics.

[10] Zhang F, Gonzalez A, Zhao M, Payne CT, Lloyd A (2003). A network of redundant bHLH proteins functions in all TTG1-dependent pathways of *Arabidopsis*. Development.

[11] Szymanski DB, Jilk RA, Pollock SM, Marks MD (1998). Control of *GL2* expression in *Arabidopsis* leaves and trichomes. Development.

[12] Exner V, Gruissem W, Hennig L (2008). Control of trichome branching by chromatin assembly factor-1. BMC Plant Biol.

[13] Perazza D, Vachon G, Herzog M (1998). Gibberellins promote trichome formation by up-regulating *GLABROUS1* in Arabidopsis. Plant Physiol.

[14] An L, Zhou Z, Su S, Yan A, Gan Y (2012). *GLABROUS INFLORESCENCE STEMS (GIS)* is required for trichome branching through gibberellic acid signaling in Arabidopsis. Plant Cell Physiol..

[15] Wei L-H, Song P, Wang Y, Lu Z, Tang Q, Yu Q (2018). The m^6^A reader ECT2 controls trichome morphology by affecting mRNA stability in Arabidopsis. Plant Cell.

[16] Szymanski DB, Lloyd AM, Marks MD (2000). Progress in the molecular genetic analysis of trichome initiation and morphogenesis in *Arabidopsis*. Trends Plant Sci.

[17] Kulich I, Vojtíková Z, Sabol P, Ortmannová J, Neděla V, Tihlaříková E, Žárský V (2018). Exocyst subunit EXO70H4 has a specific role in callose synthase secretion and silica accumulation. Plant Physiol.

[18] Kulich I, Vojtíková Z, Glanc M, Ortmannová J, Rasmann S, Žárský V (2015). Cell wall maturation of Arabidopsis trichomes is dependent on exocyst subunit EXO70H4 and involves callose deposition. Plant Physiol.

[19] Perazza D, Herzog M, Hülskamp M, Brown S, Dorne A-M, Bonneville J-M (1999). Trichome cell growth in *Arabidopsis thaliana* can be derepressed by mutations in at least five genes. Genetics.

[20] Wienkoop S, Zoeller D, Ebert B, Simon-Rosin U, Fisahn J, Glinski M, Weckwerth W (2004). Cell-specific protein profiling in *Arabidopsis thaliana* trichomes: identification of trichome-located proteins involved in sulfur metabolism and detoxification. Phytochemistry.

[21] Lieckfeldt E, Simon-Rosin U, Kose F, Zoeller D, Schliep M, Fisahn J (2008). Gene expression profiling of single epidermal, basal and trichome cells of *Arabidopsis thaliana*. J Plant Physiol.

[22] Kryvych S, Nikiforova V, Herzog M, Perazza D, Fisahn J (2008). Gene expression profiling of the different stages of *Arabidopsis thaliana* trichome development on the single cell level. Plant Physiol Biochem.

[23] Ebert B, Melle C, Lieckfeldt E, Zöller D, von Eggeling F, Fisahn J (2008). Protein profiling of single epidermal cell types from *Arabidopsis thaliana* using surface-enhanced laser desorption and ionization technology. J Plant Physiol.

[24] Ebert B, Zöller D, Erban A, Fehrle I, Hartmann J, Niehl A (2010). Metabolic profiling of *Arabidopsis thaliana* epidermal cells. J Exp Bot.

[25] Marks MD, Betancur L, Gilding E, Chen F, Bauer S, Wenger JP (2008). A new method for isolating large quantities of Arabidopsis trichomes for transcriptome, cell wall and other types of analyses. Plant J.

[26] Zhang X, Oppenheimer DG (2004). A simple and efficient method for isolating trichomes for downstream analyses. Plant Cell Physiol.

[27] Marks MD, Wenger JP, Gilding E, Jilk R, Dixon RA (2009). Transcriptome analysis of Arabidopsis wild-type and *gl3–sst sim* trichomes identifies four additional genes required for trichome development. Mol Plant.

[28] Wood PJ, Fulcher RG (1984). Specific interaction of aniline blue with (1 → 3)-β-D-glucan. Carbohydr Polym.

[29] Eschrich W, Currier HB (1964). Identification of callose by its diachrome and fluorochrome reactions. Stain Technol.

[30] Wood PJ (1980). Specificity in the interaction of direct dyes with polysaccharides. Carbohydr Res.

[31] Mitra PP, Loqué D (2014). Histochemical staining of *Arabidopsis thaliana* secondary cell wall elements. J Vis Exp.

[32] Jakoby MJ, Falkenhan D, Mader MT, Brininstool G, Wischnitzki E, Platz N (2008). Transcriptional profiling of mature Arabidopsis trichomes reveals that *NOECK* encodes the MIXTA-like transcriptional regulator MYB106. Plant Physiol.

[33] van Bel M, Diels T, Vancaester E, Kreft L, Botzki A, van de Peer Y (2018). PLAZA 4.0: an integrative resource for functional, evolutionary and comparative plant genomics. Nucleic Acids Res..

[34] Atchison DK, Beierwaltes WH (2013). The influence of extracellular and intracellular calcium on the secretion of renin. Pflugers Arch.

[35] Brownlee C (2003). Plant signalling: calcium first and second. Curr Biol.

[36] Wang S, Wang J-W, Yu N, Li C-H, Luo B, Gou J-Y (2004). Control of plant trichome development by a cotton fiber *MYB* gene. Plant Cell.

[37] Andre CM, Hausman J-F, Guerriero G (2016). *Cannabis sativa*: the plant of the thousand and one molecules. Front Plant Sci.

[38] Simmons AT, Gurr GM (2006). Trichomes of *Lycopersicon* species and their hybrids: effects on pests and natural enemies. Agric For Entomol.

[39] Cho K-S, Kwon M, Cho J-H, Im J-S, Park Y-E, Hong S-Y (2017). Characterization of trichome morphology and aphid resistance in cultivated and wild species of potato. Hortic Environ Biotechnol.

[40] Brentan Silva D, Aschenbrenner A-K, Lopes NP, Spring O (2017). Direct analyses of secondary metabolites by mass spectrometry imaging (MSI) from sunflower (*Helianthus annuus* L.) trichomes. Molecules.

[41] Uzelac B, Stojičić D, Budimir S. Glandular trichomes on the leaves of *Nicotiana tabacum*: Morphology, developmental ultrastructure, and secondary metabolites. In: Ramawat KG, Ekiert HM, Goyal S, editors. Plant cell and tissue differentiation and secondary metabolites. Cham: Springer International Publishing; 2019. p. 1–37. doi:10.1007/978-3-030-11253-0_1-1.

[42] Aschenbrenner A-K, Amrehn E, Bechtel L, Spring O (2015). Trichome differentiation on leaf primordia of *Helianthus annuus* (Asteraceae): morphology, gene expression and metabolite profile. Planta.

[43] McDowell ET, Kapteyn J, Schmidt A, Li C, Kang J-H, Descour A (2011). Comparative functional genomic analysis of *Solanum* glandular trichome types. Plant Physiol.

[44] Zhang Y, Song H, Wang X, Zhou X, Zhang K, Chen X (2020). The roles of different types of trichomes in tomato resistance to cold, drought, whiteflies, and *Botrytis*. Agronomy.

[45] Glas JJ, Schimmel BCJ, Alba JM, Escobar-Bravo R, Schuurink RC, Kant MR (2012). Plant glandular trichomes as targets for breeding or engineering of resistance to herbivores. Int J Mol Sci.

[46] Tu Y (2011). The discovery of artemisinin (qinghaosu) and gifts from Chinese medicine. Nat Med.

[47] Maleci Bini L, Giuliani C (2006). The glandular trichomes of the labiatae. A review. Acta Hortic..

[48] Schuurink R, Tissier A (2020). Glandular trichomes: micro-organs with model status?. New Phytol.

[49] Tissier A (2018). Plant secretory structures: more than just reaction bags. Curr Opin Biotechnol.

[50] Tissier A (2012). Glandular trichomes: what comes after expressed sequence tags?. Plant J.

[51] Balcke GU, Bennewitz S, Bergau N, Athmer B, Henning A, Majovsky P (2017). Multi-omics of tomato glandular trichomes reveals distinct features of central carbon metabolism supporting high productivity of specialized metabolites. Plant Cell.

[52] Balcke GU, Bennewitz S, Zabel S, Tissier A (2014). Isoprenoid and metabolite profiling of plant trichomes. Methods Mol Biol.

[53] Kundan M, Gani U, Nautiyal AK, Misra P. Molecular biology of glandular trichomes and their functions in environmental stresses. In: Molecular approaches in plant biology and environmental challenges: Springer; 2019. p. 365–393.

[54] Slocombe SP, Schauvinhold I, McQuinn RP, Besser K, Welsby NA, Harper A (2008). Transcriptomic and reverse genetic analyses of branched-chain fatty acid and acyl sugar production in *Solanum pennellii* and *Nicotiana benthamiana*. Plant Physiol.

[55] Huchelmann A, Boutry M, Hachez C (2017). Plant glandular trichomes: natural cell factories of high biotechnological interest. Plant Physiol.

[56] Spring O, Pfannstiel J, Klaiber I, Conrad J, Beifuß U, Apel L (2015). The nonvolatile metabolome of sunflower linear glandular trichomes. Phytochemistry.

[57] Huang Y, Wang L, Chao Y, Nawawi DS, Akiyama T, Yokoyama T, Matsumoto Y (2016). Relationships between hemicellulose composition and lignin structure in woods. J Wood Chem Technol.

[58] Smith LG, Hake S (1992). The initiation and determination of leaves. Plant Cell.

[59] Bischoff V, Nita S, Neumetzler L, Schindelasch D, Urbain A, Eshed R (2010). *TRICHOME BIREFRINGENCE* and its homolog *AT5G01360* encode plant-specific DUF231 proteins required for cellulose biosynthesis in Arabidopsis. Plant Physiol.

[60] Laterre R, Pottier M, Remacle C, Boutry M (2017). Photosynthetic trichomes contain a specific Rubisco with a modified pH-dependent activity. Plant Physiol.

[61] Wang X, Shen C, Meng P, Tan G, Lv L (2021). Analysis and review of trichomes in plants. BMC Plant Biol.

[62] Sinclair SA, Larue C, Bonk L, Khan A, Castillo-Michel H, Stein RJ (2017). Etiolated seedling development requires repression of photomorphogenesis by a small cell-wall-derived dark signal. Curr Biol.

[63] Pauly M, Gille S, Liu L, Mansoori N, de Souza A, Schultink A, Xiong G (2013). Hemicellulose biosynthesis. Planta.

[64] Goldberg R, Morvan C, Jauneau A, Jarvis MC. Methyl-esterification, de-esterification and gelation of pectins in the primary cell wall. In: J. Visser and A.G.J. Voragen, editor. Pectins and Pectinases: Progress in Biotechnology, Vol. 14. New York: Elsevier; 1996. p. 151–172. doi:10.1016/S0921-0423(96)80253-X.

[65] Facchini PJ (2001). Alkaloid biosynthesis in plants: biochemistry, cell biology, molecular regulation, and metabolic engineering applications. Annu Rev Plant Physiol Plant Mol Biol.

[66] Facchini PJ, Bird DA, St-Pierre B (2004). Can *Arabidopsis* make complex alkaloids?. Trends Plant Sci.

[67] Sohani MM, Schenk PM, Schultz CJ, Schmidt O (2009). Phylogenetic and transcriptional analysis of a strictosidine synthase-like gene family in *Arabidopsis thaliana* reveals involvement in plant defence responses. Plant Biol (Stuttg).

[68] Devoto A, Ellis C, Magusin A, Chang H-S, Chilcott C, Zhu T, Turner JG (2005). Expression profiling reveals *COI1* to be a key regulator of genes involved in wound- and methyl jasmonate-induced secondary metabolism, defence, and hormone interactions. Plant Mol Biol.

[69] Karabourniotis G, Liakopoulos G, Nikolopoulos D, Bresta P (2020). Protective and defensive roles of non-glandular trichomes against multiple stresses: structure–function coordination. J For Res.

[70] Conneely LJ, Mauleon R, Mieog J, Barkla BJ, Kretzschmar T (2021). Characterization of the *Cannabis sativa* glandular trichome proteome. PLoS ONE.

[71] Takemori A, Nakashima T, Ômura H, Tanaka Y, Nakata K, Nonami H, Takemori N (2019). Quantitative assay of targeted proteome in tomato trichome glandular cells using a large-scale selected reaction monitoring strategy. Plant Methods.

[72] Champagne A, Boutry M (2017). A comprehensive proteome map of glandular trichomes of hop (*Humulus lupulus* L.) female cones: Identification of biosynthetic pathways of the major terpenoid-related compounds and possible transport proteins. Proteomics.

[73] Wu T, Wang Y, Guo D (2012). Investigation of glandular trichome proteins in *Artemisia annua* L. using comparative proteomics. PLoS ONE..

[74] Roka L, Koudounas K, Daras G, Zoidakis J, Vlahou A, Kalaitzis P, Hatzopoulos P (2018). Proteome of olive non-glandular trichomes reveals protective protein network against (a)biotic challenge. J Plant Physiol.

[75] Sambrook J, Fritsch EF, Maniatis T (1989). Molecular cloning: a laboratory manual.

[76] Yeats T, Vellosillo T, Sorek N, Ibáñez AB, Bauer S (2016). Rapid determination of cellulose, neutral sugars, and uronic acids from plant cell walls by one-step two-step hydrolysis and HPAEC-PAD. Bio Protoc.

[77] Voegele RT, Schmid A (2011). RT real-time PCR-based quantification of *Uromyces fabae* in planta. FEMS Microbiol Lett.

[78] Schmittgen TD, Livak KJ (2008). Analyzing real-time PCR data by the comparative C_T_ method. Nat Protoc.

[79] Hughes CS, Moggridge S, Müller T, Sorensen PH, Morin GB, Krijgsveld J (2019). Single-pot, solid-phase-enhanced sample preparation for proteomics experiments. Nat Protoc.

[80] Rappsilber J, Mann M, Ishihama Y (2007). Protocol for micro-purification, enrichment, pre-fractionation and storage of peptides for proteomics using StageTips. Nat Protoc.

[81] Beck S, Michalski A, Raether O, Lubeck M, Kaspar S, Goedecke N (2015). The impact II, a very high-resolution quadrupole time-of-flight instrument (QTOF) for deep shotgun proteomics. Mol Cell Proteomics.

[82] Tyanova S, Temu T, Cox J (2016). The MaxQuant computational platform for mass spectrometry-based shotgun proteomics. Nat Protoc.

[83] Wickham H, Averick M, Bryan J, Chang W, McGowan L, François R (2019). Welcome to the Tidyverse. J Open Source Softw.

[84] Tyanova S, Temu T, Sinitcyn P, Carlson A, Hein MY, Geiger T (2016). The Perseus computational platform for comprehensive analysis of (prote)omics data. Nat Methods.

[85] Perez-Riverol Y, Csordas A, Bai J, Bernal-Llinares M, Hewapathirana S, Kundu DJ (2019). The PRIDE database and related tools and resources in 2019: improving support for quantification data. Nucleic Acids Res.

