# Supplementary material for: An advanced method for the release, enrichment and purification of high-quality Arabidopsis thaliana rosette leaf trichomes enables profound insights into the trichome proteome
Source: Plant Methods. 2022 Jan 28;18:12. doi: 10.1186/s13007-021-00836-0 (PMC8796501; doi:10.1186/s13007-021-00836-0)
Supplement: Supplementary file 1 — Additional file 1: Figure S1. Comparison of EGTA and EDTA as chelating agents for trichome release. Figure S2. Trichome types in RAW samples of S. lycopersicum and N. benthamiana. Figure S3. Microscopic evaluation of histochemical staining of A. thaliana wild-type trichomes with Toluidine Blue O or by Mäule reaction. Figure S4. Comparison of the densities of sucrose and Nycodenz for DGC. Figure S5. Verification of amplicon sequence integrity after qRT-PCR. Figure S6. GO terms associated with proteins enriched in trichomes. Figure S7. GO terms associated with proteins depleted in trichomes. Table S1. Trichome yield per harvested plant fresh mass. Table S2. GO terms and key words used as filter criteria to identify proteins of similar function among trichome and/or leaf samples. Table S3. A. thaliana neutral monosaccharide and cellulose amounts measured in various studies. Table S4. Stains, incubation times and illumination procedures used to probe cell wall components and lipids of trichomes. Table S5. Primers used in this study. [file 13007_2021_836_MOESM1_ESM.docx]

**
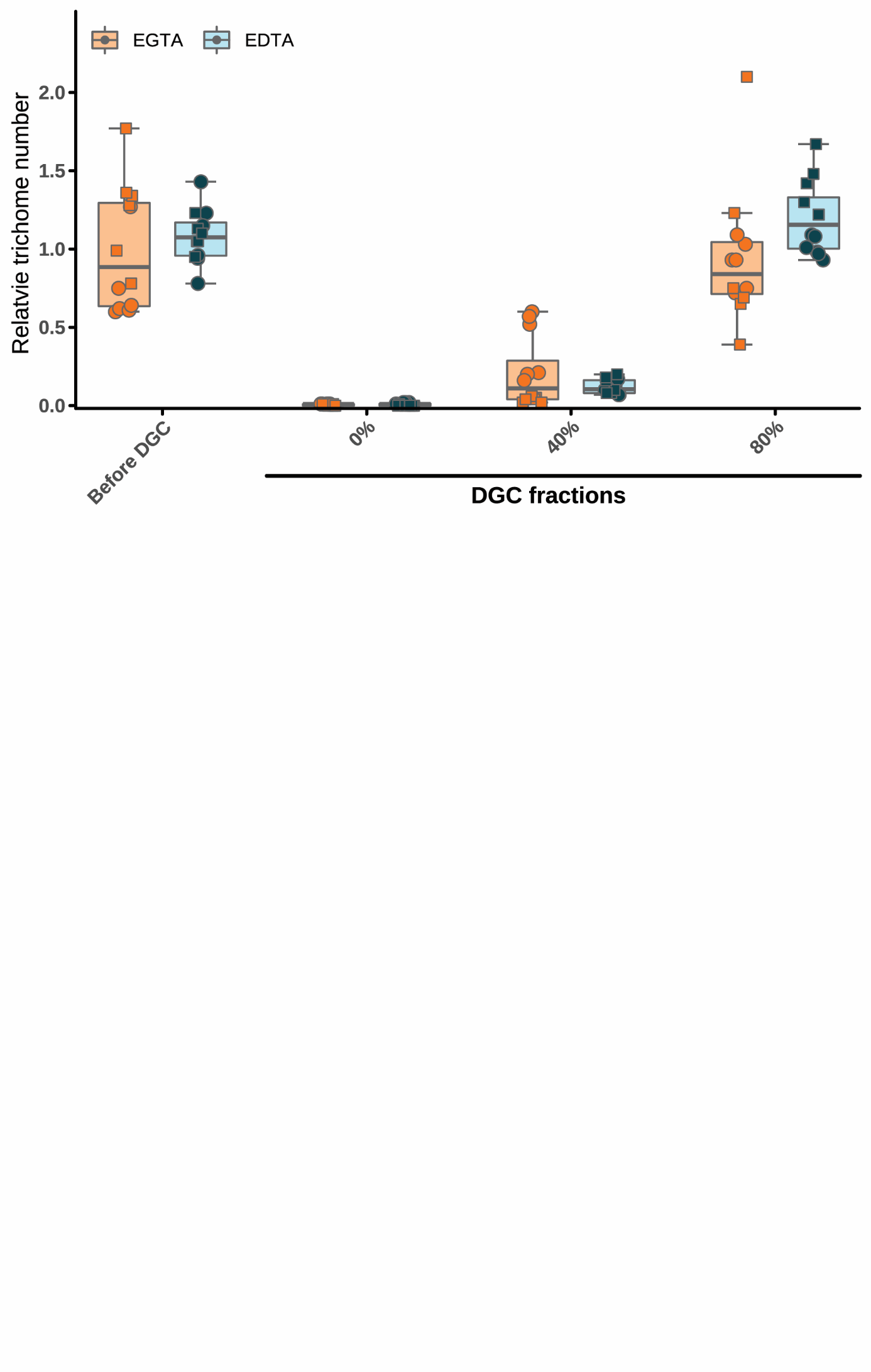
**

**Figure S1 Comparison of EGTA and EDTA as chelating agents for trichome release.** Quantification of trichomes processed by the STIRRER method using EGTA- or EDTA-containing PBS buffer for release. Trichome number is depicted relative to the mean count of Before DGC EGTA samples. The boxplot indicates the outcome of two independent experiments with six samples per experiment. Different shapes of data points mark independent experiments.


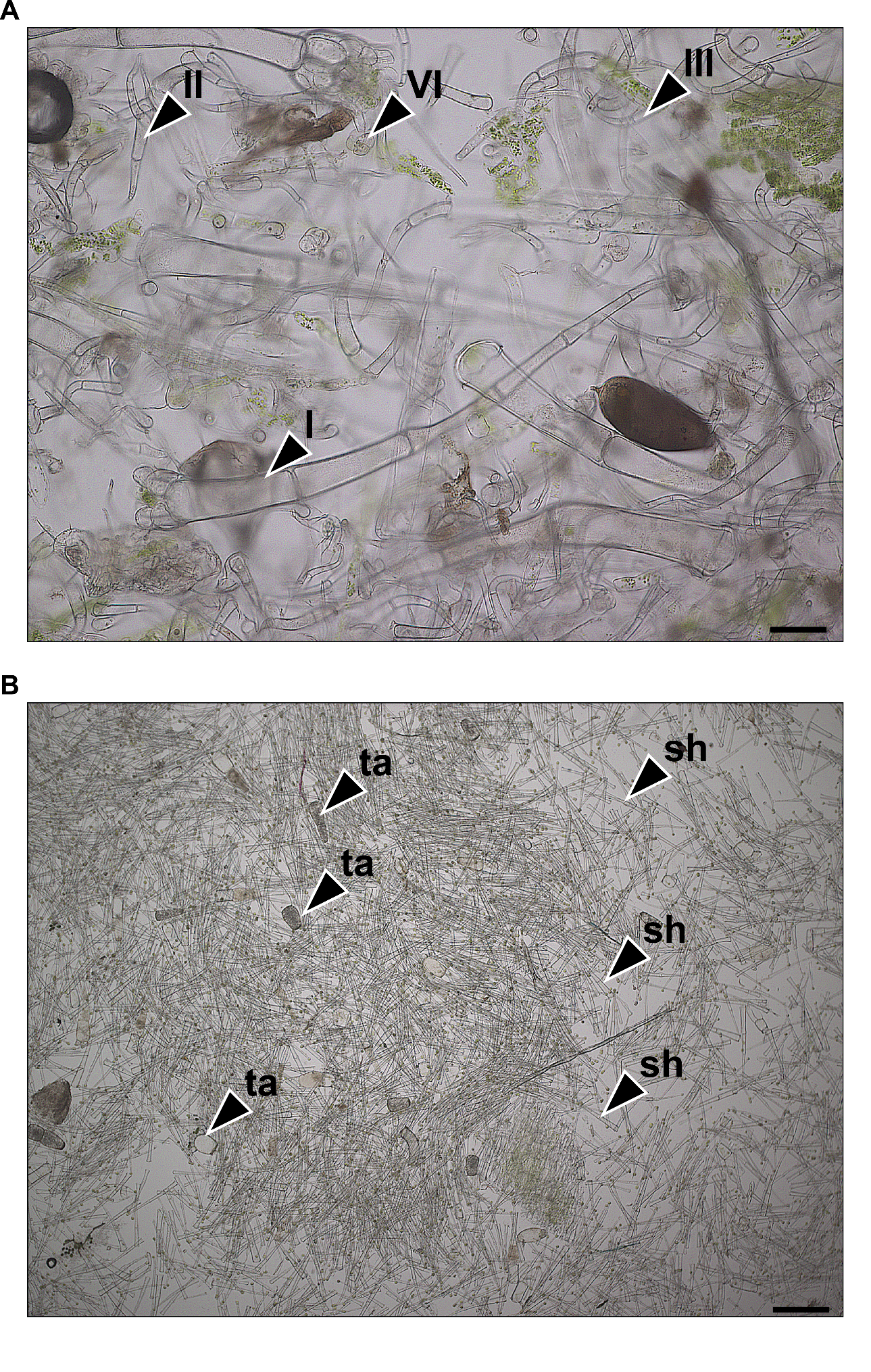


**Figure S2 Trichome types in RAW samples of *S. lycopersicum* and *N. benthamiana*. A** *S. lycopersicum* trichomes after release and enrichment. Roman numerals indicate trichome types as described in the literature. Scale bar represents 100 µm. **B** *N. benthamiana* trichomes after release and enrichment. Glandular trichomes were classified into tall (ta) and short (sh). Scale bar represents 500 µm.


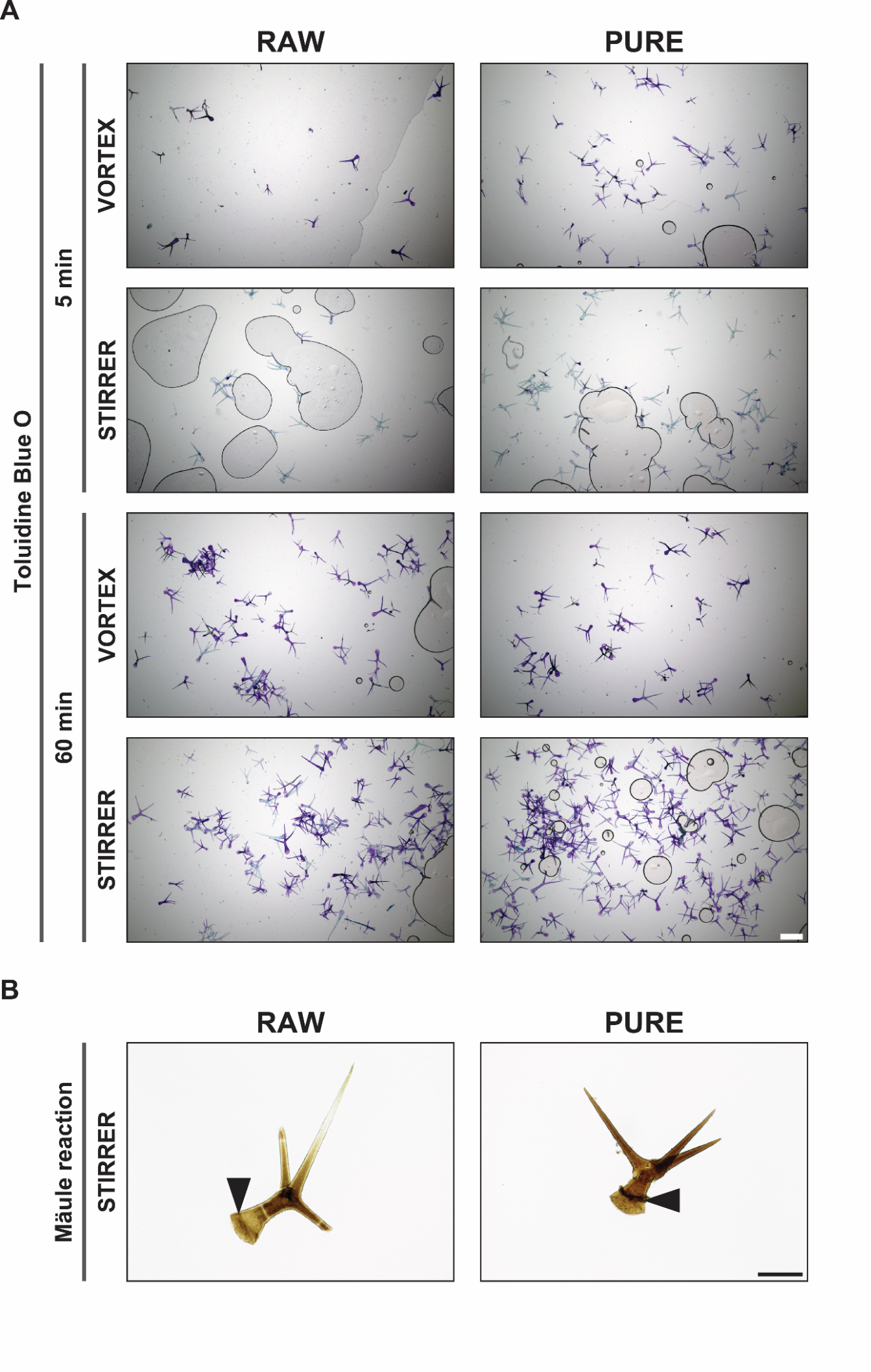


**Figure S3** **Histochemical staining of *A. thaliana* wild type trichomes and microscopic evaluation.** Isolated trichomes were subjected to histochemical staining and observation by brightfield microscopy. **A** The size bar (PURE, 60 min, STIRRER) equals 500 µm. **B** Arrowheads mark the Ortmannian ring. The size bar (PURE) equals 100 µm.


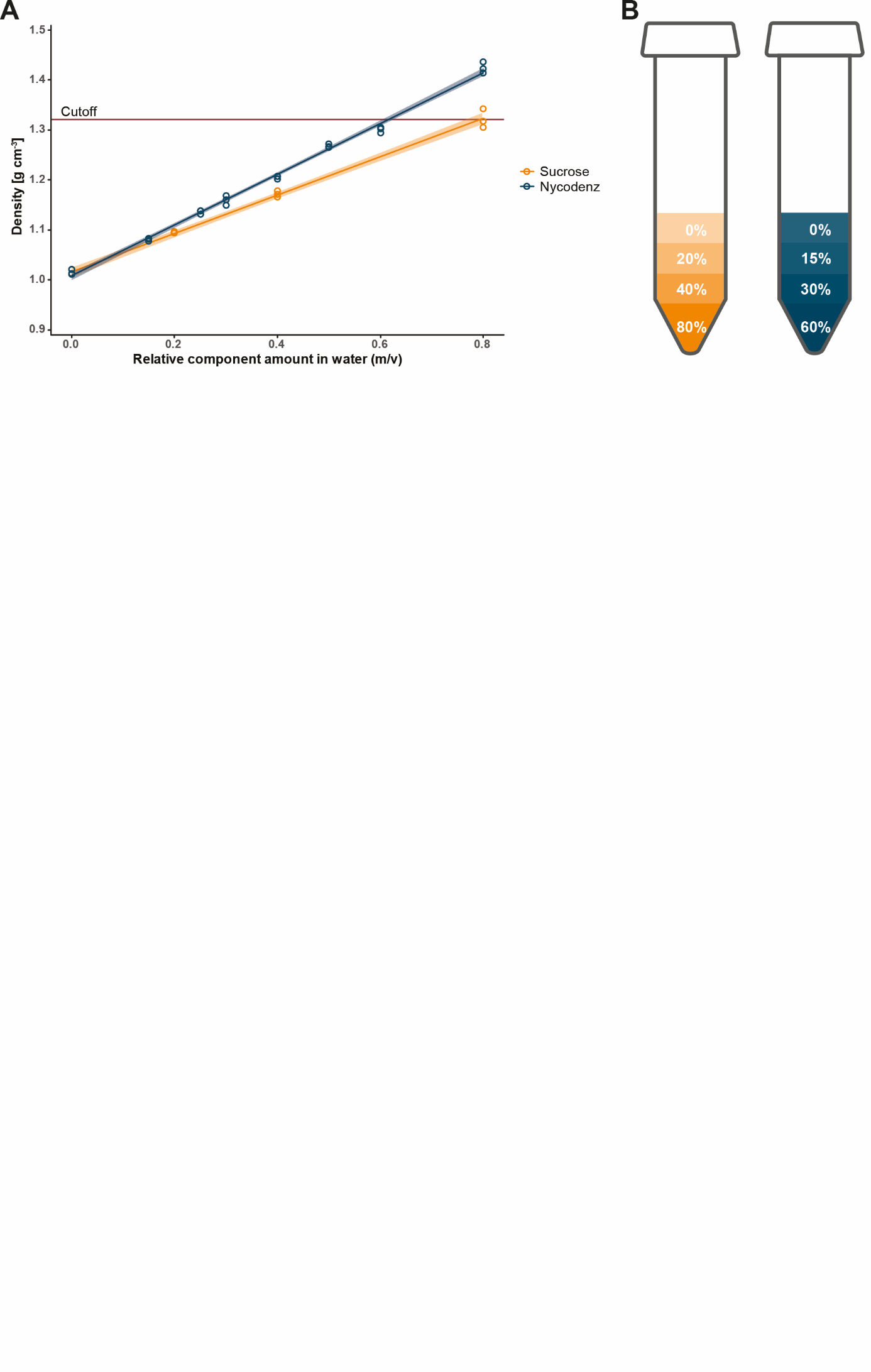


**Figure S4 Comparison of the densities of sucrose and Nycodenz for DGC. A** Masses of 200 µL sucrose or Nycodenz solutions were measured in triplicates and densities were calculated (T = 25 °C). Lines represent best fit to estimate densities of sucrose (y = 0.38x + 1.02, R2 = 0.9998) or Nycodenz (y = 0.51x + 1.01, R2 = 0.9968) within the covered concentration domain (0–80%). Horizontal line marks the cutoff for the separation of crude plant debris and trichomes. **B** Schematic depiction of sucrose or Nycodenz density gradients in a 15-mL reaction tube for trichome purification. According to our data, Nycodenz densities resembled sucrose densities upon a reduction of Nycodenz concentrations by 20% compared to sucrose.

**
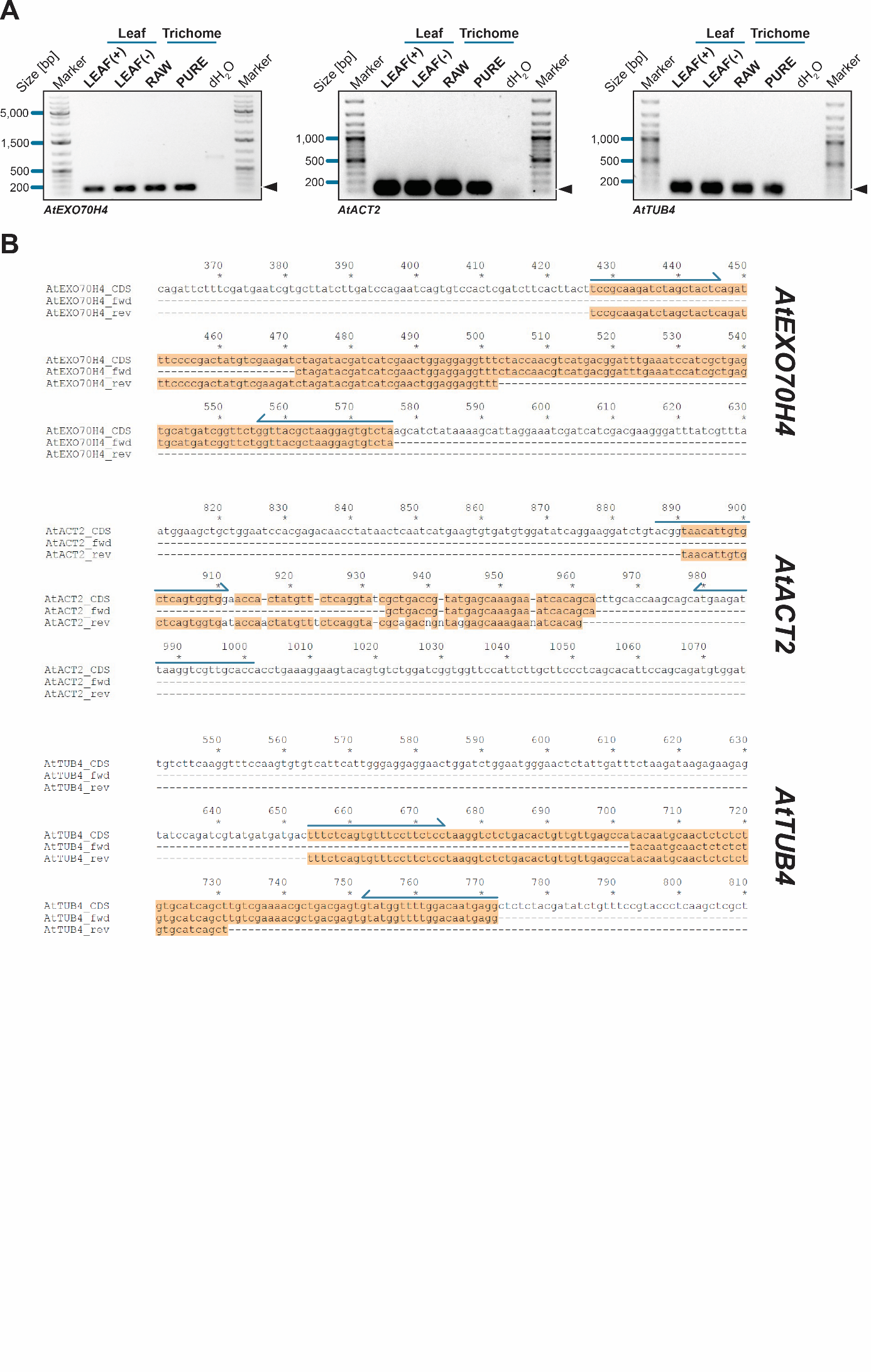
**

**Figure S5 Verification of amplicon integrity after qRT-PCR.** **A** Agarose gel electrophoresis of qRT-PCR amplicons. Arrowheads indicate expected fragment sizes. **B** Alignment of coding regions (CDS) of *AtEXO70H4* (*AT3G09520*), *AtACT2* (*AT3G18780*) and *AtTUB4* (*AT5G44340*) with sequences obtained from Sanger sequencing of qRT-PCR amplicons using the respective qRT-PCR forward or reverse primers. Blue arrows cover regions of primer binding. LEAF(+): *A. thaliana* leaves harboring trichomes, LEAF(-): Leaves after trichome release, RAW: Trichomes before DGC, PURE: Trichomes after sucrose DGC.


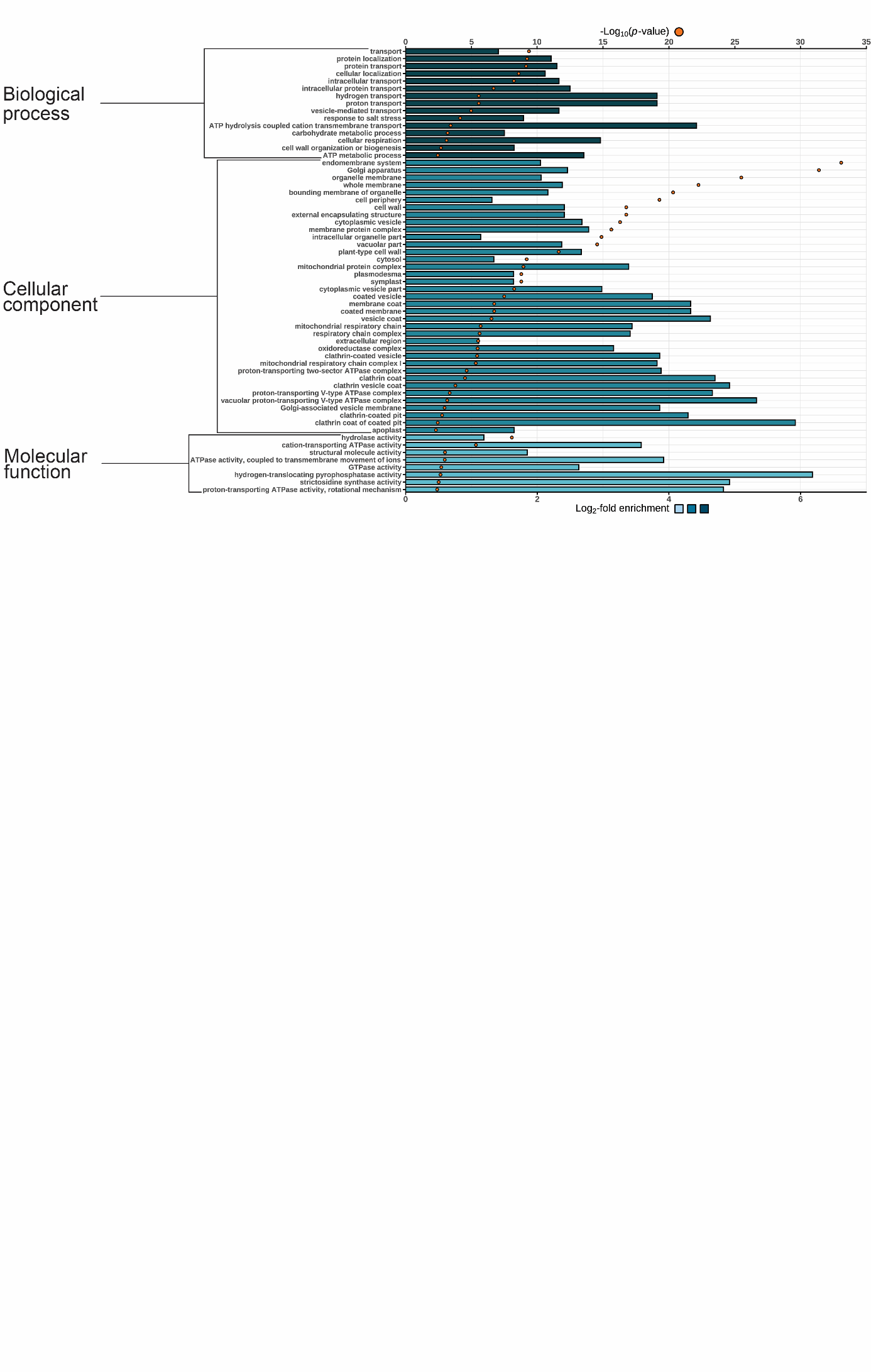


**Figure S6** **GO terms associated with proteins enriched in trichomes.** GO term enrichment was computed using PLAZA 4.5 as described in the Methods section. Bars indicate fold enrichment (lower x-axis) of GO terms grouped by biological process, cellular component and molecular function. Orange dots mark *p*-values (upper x-axis).


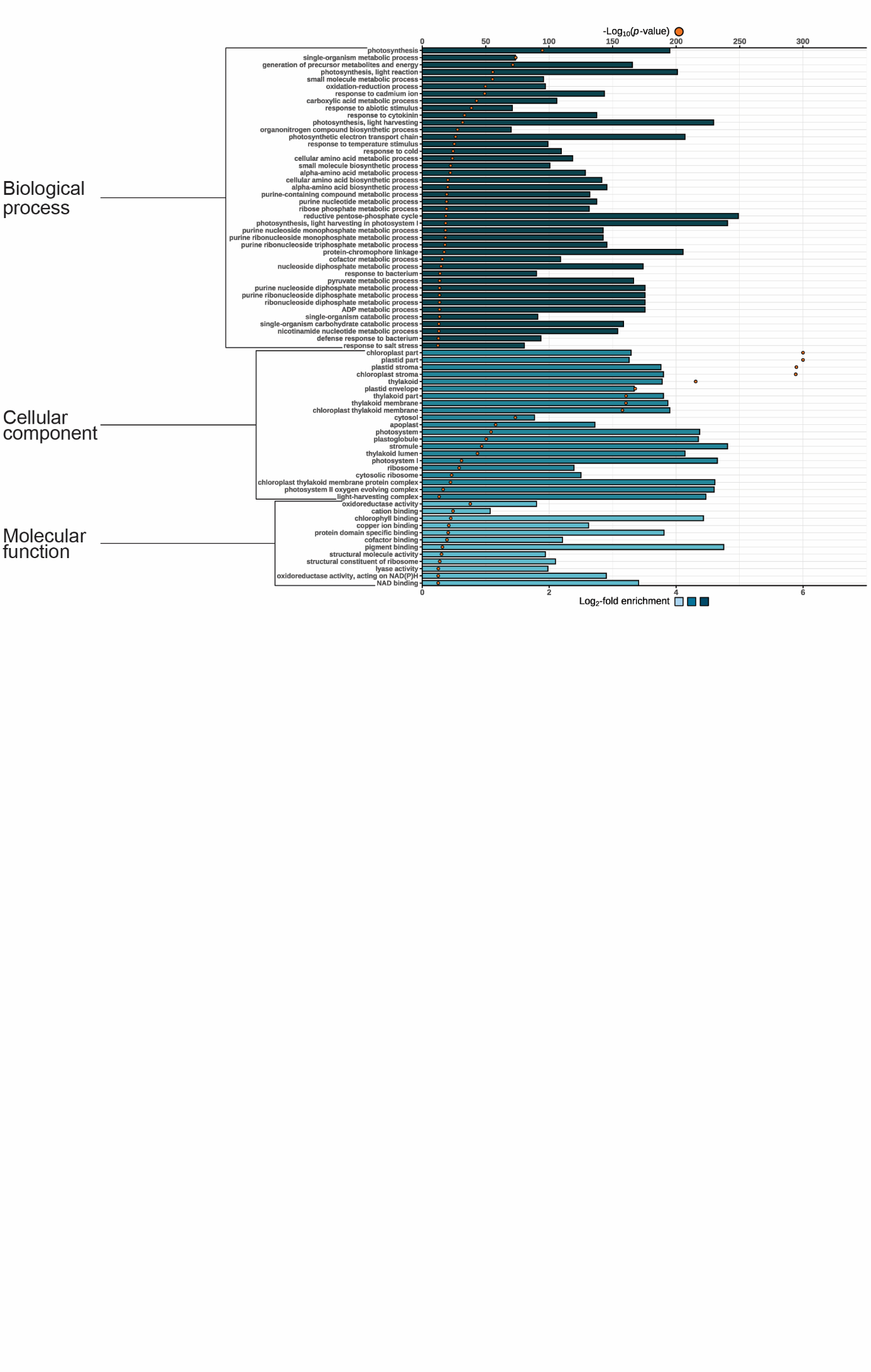


**Figure S7 GO terms associated with proteins depleted in trichomes.** GO-term enrichment was computed using PLAZA 4.5 as described in the methods section. Bars indicate fold enrichment of GO-terms grouped by biological process, cellular component and molecular function. Orange dots mark *p*-values. Plot contains the 75 most significantly enriched GO terms associated with proteins depleted in trichomes.

**Table S1 Trichome yield per harvested plant fresh mass.** Trichome bulk mass was measured before (FM) and after (DM) lyophilization. Relative trichome amounts show trichome yield per harvested plant mass.

| **Organism** | **FM_Plant_**  [g] | **FM_Trichome_**  [mg] | **DM_Trichome_**  [mg] | **Rel. FM_Trichome_**  [mg g^-1^] | **Rel. DM_Trichome_**  [mg g^-1^] |
| --- | --- | --- | --- | --- | --- |
| *A. thaliana* | ~ 36 | ~ 300 | ~ 20 | 8.33 | 0.56 |
| *S. lycopersicum* | ~ 15 | ~ 275 | ~ 16 | 18.33 | 1.07 |
| *S. tuberosum* | ~ 15 | ~ 15 | ~ 1 | 1.00 | 0.07 |
| *N. benthamiana* | ~ 15 | ~ 285 | ~ 17 | 19.00 | 1.13 |
| *H. annuus* | ~ 18 | ~ 70 | ~ 5 | 3.89 | 0.28 |

**FM**: fresh mass, **DM**: dry mass, **Rel**.: relative

**Table S2 GO terms and key words used as filter criteria to identify proteins of similar function among trichome and/or leaf samples.**

| **Group** | **Key word/term** | **Identifier** |
| --- | --- | --- |
| Sulfur metabolism and detoxification | S-adenosylmethionine biosynthetic process | GO:0006556 |
|  | methionine adenosyltransferase activity | GO:0004478 |
|  | methionine biosynthetic process | GO:0009086 |
|  | 5-methyltetrahydropteroyltriglutamate-homocysteine S-methyltransferase activity | GO:0003871 |
|  | methionine synthase activity | GO:0008705 |
|  | glutathione catabolic process | GO:0006751 |
|  | response to toxic substance | GO:0009636 |
|  | xenobiotic metabolic process | GO:0006805 |
|  | glutathione gamma-glutamylcysteinyltransferase activity | GO:0016756 |
|  | glutathione hydrolase activity | GO:0036374 |
| Pectin biosynthesis and turnover | pectin catabolic process | GO:0045490 |
|  | pectinesterase activity | GO:0030599 |
|  | pectinesterase inhibitor activity | GO:0046910 |
|  | polygalacturonase activity | GO:0004650 |
|  | pectin acetylesterase activity | GO:0052793 |
|  | O-acetyltransferase activity | GO:0016413 |
|  | cell wall pectin biosynthetic process | GO:0052325 |
|  | pectin biosynthetic process | GO:0045489 |
|  | homogalacturonan biosynthetic process | GO:0010289 |
|  | pectin metabolic process | GO:0045489 |
| Cell wall | cell wall modification | GO:0009827 |
|  | cell wall organization | GO:0071555 |
|  | plant-type cell wall | GO:0009505 |
| Golgi apparatus and vesicle | Golgi apparatus | GO:0005794 |
|  | cytoplasmic vesicle | GO:0031410 |
|  | coated vesicle |  |
|  | vesicle coat |  |
|  | clathrin-coated vesicle | GO:0030136 |
|  | clathrin coat |  |
|  | coated pit |  |
|  | vesicle-mediated transport | GO:0016192 |
| Photosynthesis | photosynthesis | GO:0015979 |

**Table S3 *A. thaliana* neutral monosaccharide and cellulose amounts measured in various studies.**

| **Study** | This study | | Marks *et al.*  (2008) | Yeats *et al.*  (2016) | Delgado-Cerezo *et al.*  (2012) | Engelsdorf *et al.*  (2019) | |
| --- | --- | --- | --- | --- | --- | --- | --- |
| **Tissue** | Trichome  (VORTEX) | Trichome  (STIRRER) | Trichome | Seedling | Leaf | Leaf | Stem |
| **Age** [d] | 42 | 42 | 28 | 5 | 21 | 35 | 35 |
| **Rhamnose**  [µg mg^-1^] | 8 | 10 | 14 | 20 | **-** | 7 | 3 |
| **Fucose**  [µg mg^-1^] | 5 | 6 | 2 | 4 | **-** | 2 | 1 |
| **Arabinose**  [µg mg^-1^] | 29 | 29 | 30 | 29 | **-** | 14 | 8 |
| **Xylose**  [µg mg^-1^] | 24 | 26 | 12 | 27 | **-** | 16 | 48 |
| **Mannose**  [µg mg^-1^] | 17 | 15 | 22 | 7 | **-** | 5 | 8 |
| **Galactose**  [µg mg^-1^] | 43 | 37 | 33 | 64 | **-** | 13 | 7 |
| **Glucose**  [µg mg^-1^] | 60 | 40 | 34 | 15 | **-** | 5 | 10 |
| **Cellulose**  [µg mg^-1^] | 332 | 315 | **-** | 128 | 100 | 170 | 340 |

References

**Delgado-Cerezo, M., Sánchez-Rodríguez, C., Escudero, V., Miedes, E., Fernández, P.V., Jordá, L., Hernández-Blanco, C., Sánchez-Vallet, A., Bednarek, P., Schulze-Lefert, P., Somerville, S., Estevez, J.M., Persson, S. and Molina, A.** (2012) Arabidopsis heterotrimeric G-protein regulates cell wall defense and resistance to necrotrophic fungi. *Mol. Plant*, 5, 98–114.

**Engelsdorf, T., Kjaer, L., Gigli-Bisceglia, N., Vaahtera, L., Bauer, S., Miedes, E., Wormit, A., James, L., Chairam, I., Molina, A. and Hamann, T.** (2019) Functional characterization of genes mediating cell wall metabolism and responses to plant cell wall integrity impairment. *BMC Plant Biology*, 19, 320.

**Marks, M.D., Betancur, L., Gilding, E., Chen, F., Bauer, S., Wenger, J.P., Dixon, R.A. and Haigler, C.H.** (2008) A new method for isolating large quantities of Arabidopsis trichomes for transcriptome, cell wall and other types of analyses. *Plant J.*, 56, 483–492.

**Yeats, T., Vellosillo, T., Sorek, N., Ibáñez, A.B. and Bauer, S.** (2016) Rapid determination of cellulose, neutral sugars, and uronic acids from plant cell walls by one-step two-step hydrolysis and HPAEC-PAD. *Bio Protoc.*, 6, e1978-e1978.

**Table S4 Stains, incubation times and illumination procedures used to probe cell wall components and lipids of trichomes.**

| **Stain** | **Incubation time** [min] | **Illumination** |
| --- | --- | --- |
| Aniline Blue | 60 | UV |
| Calcofluor White | 60 | UV |
| Ruthenium Red | 60 | Brightfield |
| Sudan Black B | 60 | Brightfield |
| Toluidine Blue O | 5/60 | Brightfield |
| Mäule | 120 | Brightfield |

**Table S5 Primers used in this study.**

| **Gene** | **Primer** | **Sequence (5’–3’)** | **T_m_** [°C] | **Amplicon size** [bp] |
| --- | --- | --- | --- | --- |
| *AtEXO70H4* | forward | TCCGCAAGATCTAGCTACTC | 60 | 149 |
|  | reverse | AGACACTCCTTAGCGTAACC | 60 |  |
| *AtACT2* | forward | GGTAACATTGTGCTCAGTGGTGG | 65 | 113 |
|  | reverse | GGTGCAACGACCTTAATCTTCAT | 63 |  |
| *AtTUB4* | forward | TTTCTCAGTGTTTCCTTCTCC | 59 | 119 |
|  | reverse | CCTCATTGTCCAAAACCATAC | 58 |  |

T_m_: Melting temperature
